# TCF/Lef regulates the Gsx ParaHox gene in central nervous system development in chordates

**DOI:** 10.1186/s12862-016-0614-3

**Published:** 2016-03-03

**Authors:** Myles G. Garstang, Peter W. Osborne, David E. K. Ferrier

**Affiliations:** The Scottish Oceans Institute, Gatty Marine Laboratory, University of St Andrews, East Sands, St Andrews, Fife KY16 8LB UK

**Keywords:** Amphioxus, *Branchiostoma floridae*, *Ciona intestinalis*, Gsx, ParaHox, Cis-regulatory element

## Abstract

**Background:**

The ParaHox genes play an integral role in the anterior-posterior (A-P) patterning of the nervous system and gut of most animals. The ParaHox cluster is an ideal system in which to study the evolution and regulation of developmental genes and gene clusters, as it displays similar regulatory phenomena to its sister cluster, the Hox cluster, but offers a much simpler system with only three genes.

**Results:**

Using *Ciona intestinalis* transgenics, we isolated a regulatory element upstream of *Branchiostoma floridae Gsx* that drives expression within the central nervous system of *Ciona* embryos. The minimal amphioxus enhancer region required to drive CNS expression has been identified, along with surrounding sequence that increases the efficiency of reporter expression throughout the *Ciona* CNS. TCF/Lef binding sites were identified and mutagenized and found to be required to drive the CNS expression. Also, individual contributions of TCF/Lef sites varied across the regulatory region, revealing a partial division of function across the Bf-Gsx-Up regulatory element. Finally, when all TCF/Lef binding sites are mutated CNS expression is not only abolished, but a latent repressive function is also unmasked.

**Conclusions:**

We have identified a *B. floridae Gsx* upstream regulatory element that drives CNS expression within transgenic *Ciona intestinalis*, and have shown that this CNS expression is dependent upon TCF/Lef binding sites. We examine the evolutionary and developmental implications of these results, and discuss the possibility of TCF/Lef not only as a regulator of chordate Gsx, but as a deeply conserved regulatory factor controlling all three ParaHox genes across the Metazoa.

**Electronic supplementary material:**

The online version of this article (doi:10.1186/s12862-016-0614-3) contains supplementary material, which is available to authorized users.

## Background

The Hox/ParaHox genes are important components of animal development with widespread roles in the evolution of body plans and morphology, as well as being prominent systems in studies of the regulation of developmental control genes. Whilst most research focuses on the Hox genes, the ParaHox genes potentially have similar importance in the evolution of development. The ParaHox cluster is the evolutionary sister to the more intensively studied Hox gene cluster [1] and the two have clear similarities in the way that they are regulated. The Hox gene cluster has been the focus of much research aimed at understanding developmental gene regulation, particularly in the context of coordinated regulation of a cluster of genes [2]. The ParaHox cluster provides an alternative, related system that is much less studied than the Hox cluster, but which presents many of the same features, whilst also being simpler due to it containing only three genes rather than the 9+ genes in intact Hox clusters [1, 3]. The phenomenon of colinearity, in which genes are expressed in the same order as they occur along the chromosome, is exhibited by both Hox and ParaHox clusters, further highlighting the similar, or potentially even homologous, mechanisms involved. Studies of retinoic acid (RA) signalling across the two clusters have provided intriguing evidence towards this [4, 5], and it is likely that this pathway is working in conjunction with the modification of chromatin between active and inactive conditions [6]. Wnt genes, important signalling molecules involved in setting up the anterior-posterior (A-P) axis, are another such signal that may play a role in the regulation of both sister clusters, showing regulation of both Hox [7, 8] and ParaHox genes [9, 10].

The regulatory phenomena governing the ParaHox cluster are relatively poorly understood, in large part because the cluster has dispersed in many species examined thus far. This is not unlike some Hox “clusters”, which have also broken apart [11–16]. It has been hypothesized that the regulatory mechanisms governing the Hox and ParaHox clusters are the basis for determining whether they are conserved as ordered clusters or instead are allowed to break apart over evolutionary time [17, 18]. Long-range gene regulatory mechanisms that are responsible for the existence of Genomic Regulatory Blocks (GRBs) could be providing a functional constraint for neighbouring genes to remain clustered together [19] in both Hox and ParaHox clusters, and have been implicated in keeping the large Hox neighbourhoods of vertebrates together [20]. We still, however, have a relatively poor understanding of the regulatory mechanisms governing Hox and ParaHox genes across a range of species, which hinders us in making deductions about the ancestral mechanisms at key points in animal evolution, such as the origin of the Bilateria or the origin of the Chordata.

Current data on ParaHox regulation is largely gleaned from studies of individual ParaHox genes in vertebrate model systems, with Cdx genes by far the most intensively studied due to their interaction with prominent caudal signals such as RA [21, 22] and Wnts [23–25] during posterior patterning. These two mechanisms may even function synergistically, with Wnt and RA cooperating to activate Cdx [26, 27], perhaps in conjunction with FGF signalling [27, 28]. It has also been suggested that these pathways may allow interactions between Hox and ParaHox genes via the modification of chromatin states [29]. Whilst there is far less data on Xlox regulation, there is evidence that it is responding to similar signals as Cdx. TCF/Lef family proteins, which can act as a mediator of canonical wnt signalling, have been shown to induce the expression of Xlox [30, 31]. RA has also been shown to induce the expression of Xlox in the pancreas of vertebrates and is crucial to the proper development of this organ, in which Xlox plays a prominent role [32–34].

There are few comparisons between the regulation of ParaHox genes in invertebrates and vertebrates, with little obvious similarity between traditional model systems such as *Drosophila* and vertebrates. That is not to say, however, that there are no similarities between distant taxa. In the beetle *Tribolium castaneum wingless* (*Wnt*) regulates *Cdx* expression in the posterior growth zone [35], suggesting that the role of Wnt in regulating Cdx in the posterior may be a conserved mechanism between insects and vertebrates. In addition, Gsx has been shown to have a conserved role in the formation of a Nkx-Gsx-Msx patterning system present in both the neurectoderm of *Drosophila* [36, 37] and the neural plate of *Xenopus* [38], with Gsx possibly displaying an ancestral role in intermediate neuron development of the last common ancestor of Bilateria, although wider taxon sampling is clearly necessary [39]. Despite the scarcity of data on Gsx regulation compared to Xlox and Cdx, it is a promising candidate for observing conserved regulatory mechanisms due to its conserved expression across the Bilateria. There is strikingly similar expression between Gsx in the brain and ventral nerve cord of protostomes, in both the Ecdysozoa [37, 40] and Lophotrochozoa [14, 41, 42] and in the brain and spinal cord of vertebrates [43–47]. In the chordates specifically, there is conservation of an early ‘hindbrain’ domain between amphioxus and the vertebrates, as well as expression in the vertebrate mid/forebrain, the sensory vesicle of the tunicate *Ciona intestinalis* [48], and the cerebral vesicle of amphioxus [4]. Deep conservation of some regulatory mechanism(s) is thus a distinct possibility.

Amphioxus plays a key role in helping to elucidate the origins of the vertebrates and chordates. This is in large part due to its position as the most basal chordate lineage, its genome having retained many features of the pre-duplicative state prior to the two whole rounds of genome duplication that occurred at the origin of the vertebrates, and its archetypal chordate morphology and development [49]. The ParaHox cluster was first described in amphioxus [50] and the colinearity of the ParaHox genes is most obvious in *Branchiostoma floridae* [3], but is also present in hemichordates [16], vertebrates [45, 51, 52] and possibly echinoderms, with the sea star *Patiria miniata* also showing chordate-like ParaHox expression [15]. This places amphioxus in a unique position in which to draw from vertebrate studies and examine regulatory pathways that may have a more widely conserved role in ParaHox regulation.

*Ciona* provides a system that is highly amenable to analysis of cis-regulatory elements via embryo electroporation of reporter gene constructs [53]. Although there are some initial results illustrating that reporter gene analyses can be done in amphioxus [54–56], the technique is currently still much more challenging in this species. Cross-species transgenesis between amphioxus and *Ciona* has, however, provided an alternative route to rapidly analysing putative amphioxus regulatory elements in vivo [55, 57, 58].

Here, we use *C. intestinalis* as a system in which to test the function of amphioxus ParaHox regulatory elements using cross-species transgenesis, assessing the ability of ParaHox regulatory elements to function across chordate sub-phyla with the aim of identifying functionally conserved regulatory mechanisms. We focused on the upstream region of the ParaHox gene *Gsx* of *B. floridae* (*Bf-Gsx*) to dissect the control of ParaHox regulatory elements. Using deletion analysis along with mutagenesis of specific transcription factor binding sites, we show that TCF/Lef sites are crucial to the function of this amphioxus regulatory element within the *C. intestinalis* reporter system, driving expression in the central nervous system. In addition, mutation of these binding motifs not only abolishes regulatory element-driven expression but also unmasks a latent repressive function that actively prevents ‘leaky’ transcription associated with the LacZ reporter. We conclude that TCF/Lef is likely to be a key factor involved in the regulation of Gsx across the chordate phylum, and discuss the possibility that TCF/Lef and Wnt signalling may play deeply conserved roles in the regulation of the ParaHox genes.

## Results

### An amphioxus *Gsx* regulatory element drives expression of a LacZ reporter throughout the neural tube of *C. intestinalis*

**Fig. 1:**
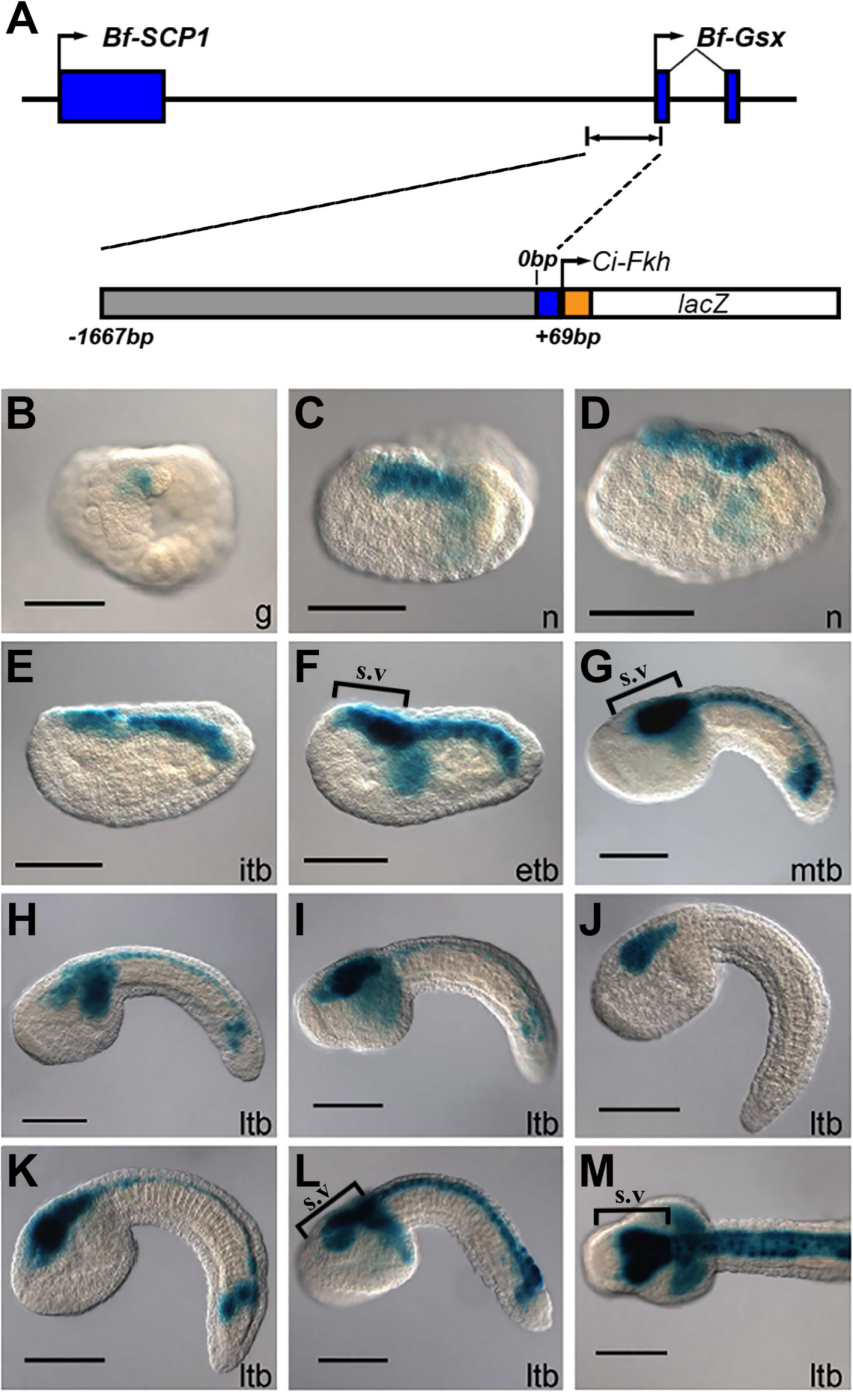
Expression of the Bf-Gsx-Up-Proximal construct in *Ciona intestinalis*. **a** Genomic map of the region comprising the Bf-Gsx-Up-Proximal regulatory element, with pCES LacZ reporter schematic. **b**-**d** LacZ expression is observed from the earliest collected stages in neural plate cells. **e**-**m** Tailbud stages: expression can be observed in the mid-posterior of the sensory vesicle, the visceral ganglion and in every cell of the tail nerve cord. Only the very anterior tip of the sensory vesicle does not express LacZ. **j** ltb embryo with expression only in the sensory vesicle. **m** Anterior region of a *Ciona* larva displaying strong expression in all four rows (*dorsal*, *ventral*, *left and right*) of the tail nerve cord. All embryos and larvae are lateral views (except B and M, which are dorsal views) with anterior to left. Square brackets in (**l**-**m**) indicate the extent of the sensory vesicle. Lower case lettering refers to the stage of development; g, gastrula; n, neurula; itb, initial tailbud; etb, early tailbud; mtb, mid tailbud; ltb, late tailbud. Scale bars represent 100 μm

To screen for potential amphioxus ParaHox gene regulatory elements we have taken advantage of the ability to rapidly transform *C. intestinalis* embryos via electroporation. A 1.7 kb upstream region of *B. floridae* Gsx, Bf-Gsx-Up-Proximal, spanning from −1667 to +69 bp from the translational start site, was cloned into the multiple cloning site of the pCES LacZ reporter (Fig. 1a) and found to reliably drive expression of LacZ throughout the central nervous system of *C. intestinalis* embryos. The Bf-Gsx-Up-Proximal driven expression throughout the central nervous system was first detected in the neural plate of early stages (Fig. 1b-e) and then throughout the neural tube, except for the most anterior region of the sensory vesicle (Fig. 1f-m). This expression was found to be highly reproducible, notwithstanding the fact that not all embryos expressed LacZ within all cells of the CNS due to the mosaic and transient nature of *C. intestinalis* electroporation-mediated transgenesis.

### Bf-Gsx-Up1c is the minimal enhancer required for nerve cord expression

**Fig. 2:**
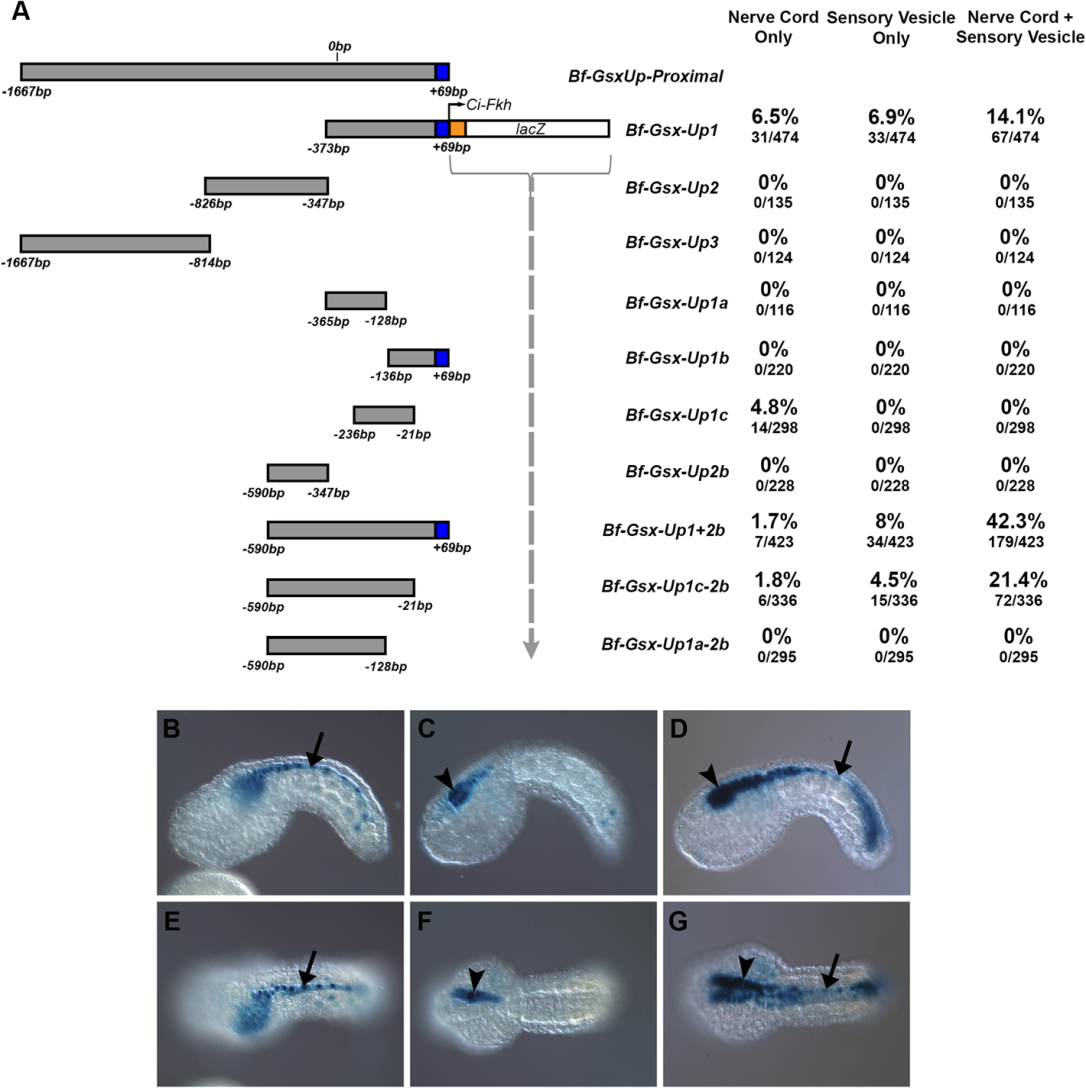
Deletion analysis of the Bf-Gsx-Up-Proximal construct. **a** Deletion map showing the deletion analysis of the Bf-Gsx-Up-Proximal construct with the numbers of embryos exhibiting LacZ expression in the nerve cord only, sensory vesicle only, or nerve cord with sensory vesicle for each construct recorded both as a percentage of the total number of embryos that developed, and as raw numbers of embryos expressing LacZ for each domain alongside the total number of embryos that developed. Grey regions indicate the relative positions of each construct compared to the Bf-Gsx-Up-Proximal construct, with 5′ and 3′ limits denoted in number of base pairs from the *B.floridae Gsx* translational start site. Blue regions denote coding sequence, whereas orange regions indicate the pCES Forkhead promoter. The Grey dashed-arrow indicates that the pCES Forkhead-LacZ construct directly abuts the displayed regulatory region in each reporter construct. **b** Lateral view of a mid tailbud *Ciona* embryo displaying ‘nerve cord only’ LacZ expression. **c** Lateral view of a mid tailbud *Ciona* embryo displaying ‘sensory vesicle only’ LacZ expression. **d** Lateral view of a mid tailbud *Ciona* embryo displaying the full ‘nerve cord with sensory vesicle’ LacZ expression pattern. **e** Dorsal view of a mid tailbud *Ciona* embryo displaying ‘nerve cord only’ LacZ expression. **f** Dorsal view of a mid tailbud *Ciona* embryo displaying ‘sensory vesicle only’ LacZ expression. **g** Dorsal view of a mid tailbud *Ciona* embryo displaying the ‘nerve cord with sensory vesicle’ LacZ expression pattern. Black arrows denote LacZ expression within the nerve cord. Black arrowheads denote LacZ expression in the sensory vesicle

From Bf-Gsx-Up-Proximal producing strong, specific neural tube expression, we used deletion analysis to find the minimal region required for neural tube expression, cutting down the original region into several smaller stretches. Three smaller, overlapping constructs were made, covering the length of the proximal region; Bf-Gsx-Up1, Bf-Gsx-Up2 and Bf-Gsx-Up3 (Fig. 2a). These were electroporated into *C. intestinalis* zygotes and expression followed through to the mid tailbud stage, where the sensory vesicle, visceral ganglion and nerve cord could be easily distinguished. Of the three, only Bf-Gsx-Up1, the most 3′ region, was able to drive expression of the LacZ reporter in the same manner as Bf-Gsx-Up-Proximal (Fig. 2a). As the Up1 region was still producing robust CNS expression it was further divided in half, into Bf-Gsx-Up1a and Bf-Gsx-Up1b, to see if we could further refine the Bf-Gsx enhancer region. Neither Up1a nor Up1b showed any CNS expression in *C. intestinalis* embryos (Fig. 2a). In order to examine if this break between 1a and 1b had disrupted a crucial element in the centre of Bf-Gsx-Up1, a further region, Bf-Gsx-Up1c, was created spanning the centre region of Up1. With this new construct, Bf-Gsx-Up1c, LacZ expression was detected in the nerve cord and visceral ganglion but not the sensory vesicle (Fig. 2a), showing it was able to function independently of the surrounding sequence, albeit at a lower efficiency. Thus, it can be concluded that Bf-Gsx-Up1c, a region of 215 bp (−236 to −21 bp from the translational start site), is the minimal regulatory region required for nerve cord expression in *C. intestinalis* embryos.

### CNS expression is dependent on the function of TCF/Lef binding sites

As neither Bf-Gsx-Up1a nor Up1b show CNS expression, yet both Up1 and Up1c do, there must be something about this central region that is crucial to the activity of this regulatory region. With expression being abolished when Up1 was split in half, we hypothesized that at least two binding sites, on either side of the Up1a/1b split, could be functioning in conjunction with one another and that when this region was broken they were not able to drive expression alone. In order to identify transcription factors that may be coordinating the function of this regulatory region, polymorphisms within the Bf-Gsx-Up regulatory region were analysed to identify conserved transcription factor binding sites (TFBs). 11 independent Bf-Gsx-Up-proximal sequences were submitted to MULAN and analysed using the multiTF program (using vertebrate TFBs) [59], identifying a series of 87 conserved potential binding sites across the Bf-Gsx-Up1 construct. These were then cross-referenced against the ANISEED database [60] to leave a list of 7 transcription factors expressed throughout the entire neural tube of *C. intestinalis*. Of these 7, there were 3 Ets binding factors (*Ci-Ets, Cin-ERF and Ets79D*), *SoxC*, *Hunchback-like*, *RAR*, and *TCF/Lef*. Of these factors, all seven are strongly expressed in other tissues that do not express the Bf-Gsx-Up construct apart from TCF/Lef.

**Fig. 3:**
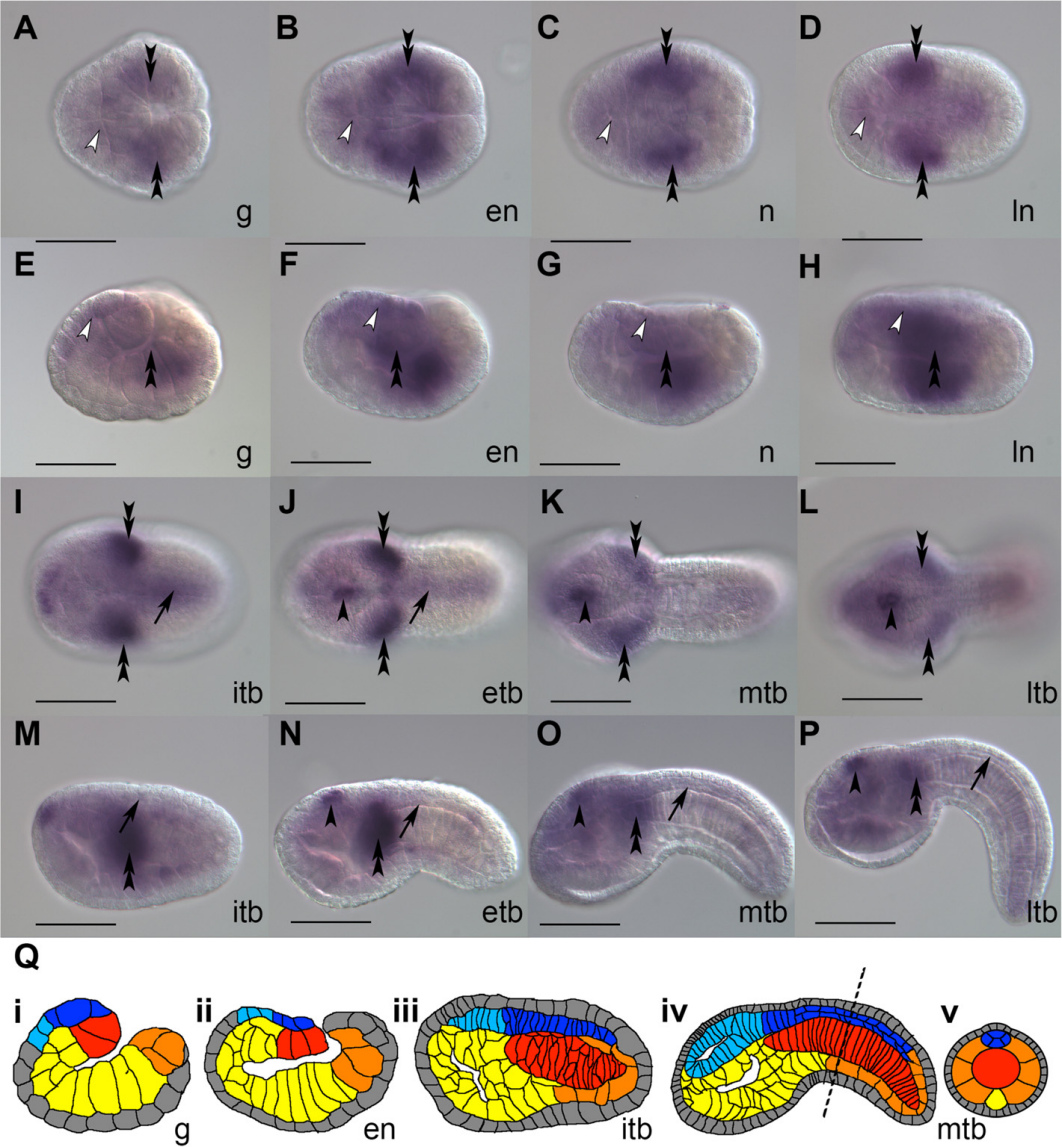
Expression of *Ciona intestinalis TCF/Lef*. In situ hybridization of *Ci-TCF/Lef* mRNA. Expression begins in mesenchymal and neural plate cells (**a**, **e**), before becoming more widespread in the neural plate (*white arrow heads*) and head/lateral trunk mesenchyme (*black double arrowheads*) (**b**, **c**, **d**, **f**, **g**, **h**), with possibly some staining also in the endoderm (**g**, **h**). In tailbud stages staining becomes more refined in the head/lateral trunk mesenchyme and weakly throughout the nerve cord (*black arrow*). Weak staining can also be seen in the endodermal strand in initial and early tailbud stages (**i**, **j**, **m**, **n**). From early tailbud onwards, a strong domain of expression exists within the centre of the sensory vesicle (*black single arrowhead*) (**j**, **k**, **l**, **n**, **o**, **p**), which remains even when staining becomes weaker in the rest of the CNS in the mid-late tailbud (**k**, **l**, **o**, **p**). **q** *i*-*v* Schematic showing cell and tissue fates through developing embryos. Schematics are made from traces of the embryos in (**e**, **f**, **m**, **o**), though numbers of cells displayed may not be absolutely accurate due to cell membranes not being visible in different focal planes. **q** *v* Represents a transverse section through the plane shown by the dotted line in (**q** *iv*). Presumptive notochord cells are shown in red, endoderm yellow, muscle orange, epidermis gray, nerve cord dark blue and sensory vesicle light blue. **a**-**d** and **i**-**l** represent dorsal views, whilst **e**-**h** and **m**-**p** represent lateral views. Lower case lettering refers to the stage of development; g, gastrula; en, early neurula; n, neurula; ln, late neurula; itb, initial tailbud; etb, early tailbud; mtb, mid tailbud; ltb, late tailbud. Scale bars represent 100 μm

To confirm the expression pattern of *C. intestinalis TCF/Lef* a time course of expression around the stages in which the Bf-Gsx-UpProximal reporter is activated was determined by whole mount in situ hybridisation with a fragment of *Ci-TCF/Lef* cDNA (Fig. 3). This time course confirms that *C. intestinalis TCF/Lef* is expressed in the developing neural plate during the mid gastrula through neurula stages (Fig. 3a-g) when the reporter is first activated, but also in the sensory vesicle and more weakly throughout the neural tube into the tailbud stages (Fig. 3h-o). *Ci-TCF/Lef* also appears to be expressed in the lateral head mesenchyme (Fig. 3d, g, h-o), which happens to correlate with a particular component of the pCES background expression that is seen in some of the reporter constructs (Additional file 1: Figure S1). This ‘background’ expression is inherent to the forkhead promoter of pCES and has been well characterised as LacZ expression in the head/neck mesenchyme and tail muscle cells (Additional file 1: Figure S1C-H ), with some animals also showing LacZ in the centre of the sensory vesicle (Additional file 1: Figure S1C, E, G, H). This background becomes much weaker or is completely abolished when a regulatory element is driving the promoter. The head/neck mesenchyme and sensory vesicle pCES background expression appears to follow a similar pattern to that of *C. intestinalis TCF/Lef*. The sensory vesicle expression that is seen as pCES background is easy to distinguish from the sensory vesicle expression seen with the Bf-Gsx-Up constructs, as the Bf-Gsx-Up regulatory elements drive expression much more extensively throughout the sensory vesicle.

**Fig. 4:**
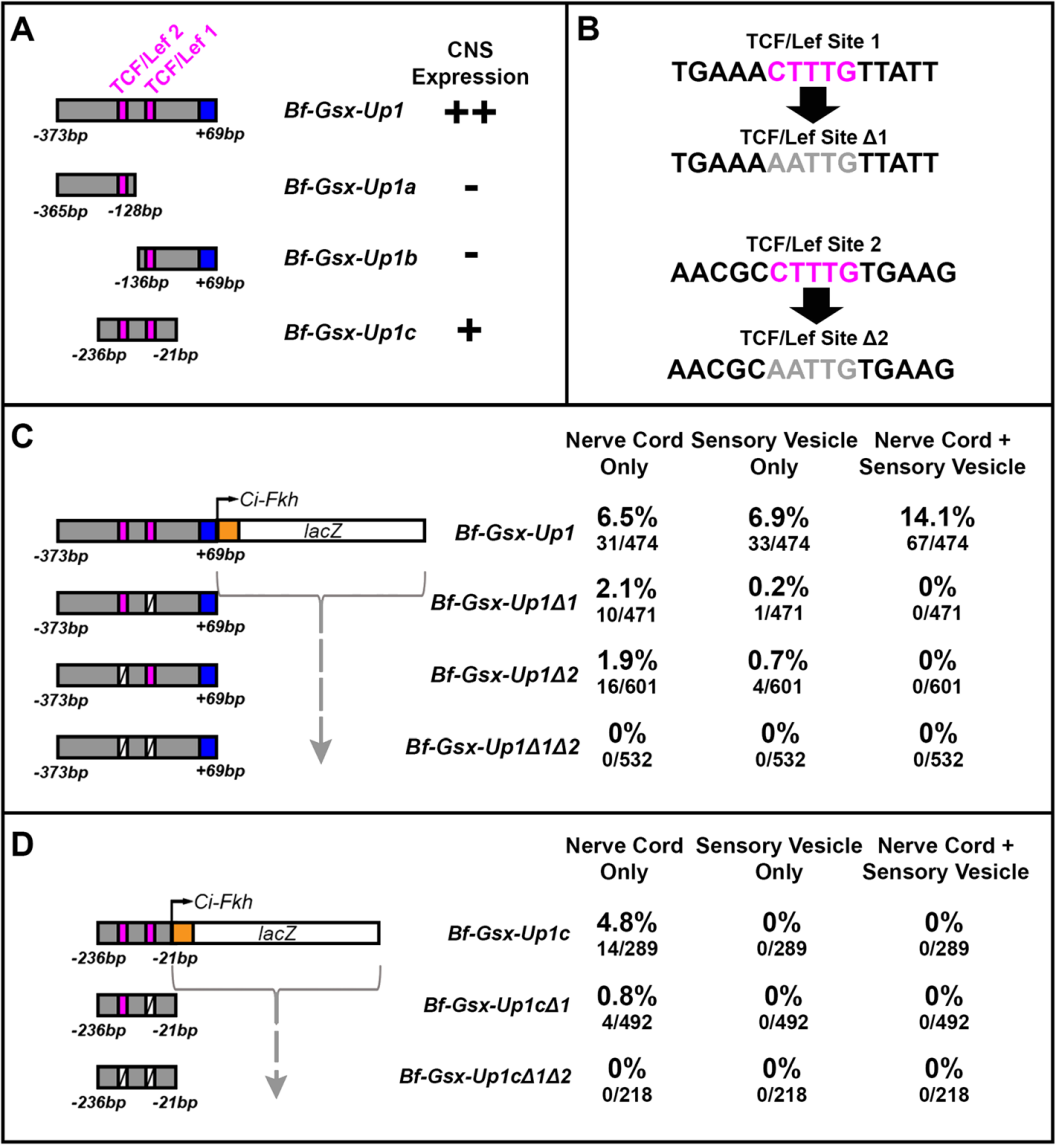
Mutation of TCF/Lef sites within the Bf-Gsx-Up1 and Bf-Gsx-Up1c constructs. **a** Relative positions of TCF/Lef sites within the Bf-Gsx-Up1, Bf-Gsx-Up1a, Bf-Gsx-Up1b and Bf-Gsx-Up1c regulatory regions. TCF/Lef sites lie either side of the Bf-Gsx-Up1a/Bf-Gsx-Up1b split. ‘+’ symbols denote the presence of LacZ expression in the corresponding construct, with ‘+’ denoting low LacZ expression and ‘++’ high LacZ expression, whereas ‘–‘ denotes the absence of LacZ expression. **b** Schematic showing the DNA sequence of TCF/Lef site1 and TCF/Lef site2 before and after mutagenesis. Pink sequence denotes the TCF/Lef site ‘core’ sequence before mutation, whereas light gray sequence denotes the TCF/Lef site ‘core’ sequence after mutation. **c** Comparison of CNS expression incidence in the Bf-Gsx-Up1 construct with TCF/Lef binding motif mutants. The numbers of embryos displaying either nerve cord only, sensory vesicle only, or the nerve cord with sensory vesicle LacZ expression patterns have been recorded both as a percentage of the total number of embryos that developed and as raw numbers of embryos expressing LacZ for each domain alongside the total number of embryos that developed. Pink boxes denote the positions of intact TCF/Lef sites, whereas white crossed boxes indicate the positions of mutated TCF/Lef sites. **d** Comparison of CNS expression incidence in the Bf-Gsx-Up1c minimal enhancer construct with TCF/Lef binding motif mutants, with annotation as for (**c**)

In further support for the role of TCF/Lef as a possible activator of the regulatory region, TCF/Lef binding sites are also located on either side of the Up1a/1b boundary, present as a pair in both Bf-Gsx-Up1 and the minimal enhancer Bf-Gsx-Up1c (Fig. 4a). These sites have been well characterised and the consensus 5′-CTTTG[A/T][A/T]-3′ is widely accepted as being TCF/Lef specific. In order to test if these sites are involved in driving CNS expression, the two sites were mutagenized in both Bf-Gsx-Up1 and the minimal enhancer Bf-Gsx-Up1c. One of these sites matches the consensus with CTTTGTT, whilst the second site has the slightly divergent CTTTGTG. This second site was not discounted as the flanking sequence shows similarity to TCF/Lef sites and it also occupies the functionally relevant location on one side of the Gsx-Up1a/1b split. Indeed, it may be that a single G has been inserted within this particular site, but has not disrupted the CTTTG core and so could remain functional.

The mutagenesis focused on the core CTTTG element of each TCF/Lef binding site, which has no redundancy in the consensus. In order to alter this core element in each site the following TCF/Lef site mutations were produced; SiteΔ1: TGAAA**AATTG**TTATT, Site Δ2: AACGC**AATTG**TGAAG (Fig. 4b). These mutations were carried out both separately and as a double TCF/Lef site mutation, with the numbers of animals expressing either nerve cord, sensory vesicle, or both nerve cord and sensory vesicle expression noted for each resulting construct (Fig. 4c). With both Bf-Gsx-Up1Δ1 and Bf-Gsx-Up1Δ2, mutation of either site alone abolishes the nerve cord with sensory vesicle expression, and reduces individual nerve cord expression, from 6.5 to 2.1 % in Δ1 and 1.9 % in Δ2, and sensory vesicle expression, from 6.9 to 0.2 % in Δ1 and 0.7 % in Δ2 (Fig. 4c). This implies that TCF/Lef sites are contributing to the expression in the CNS, and could be functioning cumulatively. With the double TCF/Lef site mutation, Bf-Gsx-UpΔ1Δ2, the CNS expression in Bf-Gsx-Up1 is completely abolished, with no animals showing either nerve cord or sensory vesicle expression, confirming that these TCF/Lef sites are crucial to the function of the regulatory region (Fig. 4c). In order to test if the minimal enhancer, Bf-Gsx-Up1c, is indeed functioning as suspected by allowing these two TCF/Lef sites to interact and drive nerve cord expression, the same Δ1 and Δ2 mutations were introduced into the minimal enhancer region. Again, numbers of embryos expressing LacZ in either nerve cord alone, sensory vesicle alone, or in nerve cord and sensory vesicle were noted (Fig. 4d). Both Bf-Gsx-Up1cΔ1 and Bf-Gsx-Up1cΔ1Δ2 were cloned into pCES successfully, but unfortunately Bf-Gsx-Up1cΔ2 was refractory to cloning into pCES. Nevertheless, as with Bf-Gsx-Up1, the single site mutation of Bf-Gsx-Up1Δ1 reduces efficiency of nerve cord expression (Fig. 4d). Again, the double mutation in Bf-Gsx-Up1cΔ1Δ2 completely abolishes nerve cord expression for this minimal region as it does in the larger Bf-Gsx-Up1 (Fig. 4c, d).

### Addition of the Bf-Gsx-2b region increases expression efficiency, but still requires the Bf-Gsx-Up1c region in order to drive CNS expression

The multiple sequence alignments that indicate the levels of polymorphism across this Bf-Gsx regulatory region (Additional file 2: Figure S2), in addition to helping identify conserved TFBs, also identified highly conserved regions outside of Bf-Gsx-Up1 that could potentially also contribute to expression. This region of conservation also extends to a second species of amphioxus, *Branchiostoma belcheri* [61, 62] (Additional file 2: Figure S2). The 11 *B. floridae* Gsx-UpProximal sequences and single *B. belcheri* Gsx-UpProximal sequence were aligned and visualised using VISTA [63] to identify regions of high/low polymorphism across the regulatory region (Additional file 2: Figure S2). As expected, the Gsx-Up1 region was highly conserved across *B. floridae* individuals, but this conservation also extended into the 3′ half of Gsx-Up2, perhaps indicating that this sequence may also contribute to the function of the region. Interestingly, this same pattern of conservation is more apparent when comparing between amphioxus species, *B. floridae* and *B. belcheri* (Additional file 2: Figure S2). The same is also true for *B. lanceolatum* (unpublished data). This cross-species analysis also highlights a drop off in sequence conservation at the 3′ of the Up1 region, which correlates with the 3′ end of the Up1c minimal enhancer region.

In order to examine the function of this proximal region that extends into Bf-Gsx-Up2, a longer construct was produced, Bf-Gsx-Up1 + 2b (−590 to +69 bp). When electroporated, this region produced lower numbers of individuals with nerve cord only expression, at 1.7 % (down from 6.5 % with Bf-Gsx-Up1), similar numbers of individuals with only sensory vesicle expression (8 % compared to 6.9 %), whilst having drastically increased numbers of animals showing the nerve cord with sensory vesicle expression, up from 14.1 % in Bf-Gsx-Up1 to 42.3 % of embryos in Bf-Gsx-Up1 + 2b (Fig. 2a). This increase in the prevalence of full CNS expression suggested that the additional conserved sequence is in fact important to the function of the regulatory region, in contrast to the results suggested by the lack of CNS expression in the Bf-Gsx-Up2 construct alone (Fig. 2a). To identify if this expanded region was still dependant on the minimal enhancer, two more constructs were made, to test whether a region important for CNS expression had been split when making Bf-Gsx-Up1 and -Up2, as had happened with Bf-Gsx-Up1a and -Up1b. The first construct, Bf-Gsx-Up1c-2b (−590 to −21 bp), was identical to Bf-Gsx-Up1 + 2b in all respects except that it stopped at the 3′ boundary of the Up1c region. This construct still produced the full nerve cord plus sensory vesicle expression seen in both Bf-Gsx-Up-Proximal and Bf-Gsx-Up1, at higher numbers than Bf-Gsx-Up1 (21.4 % up from 14.1 % in Bf-Gsx-Up1), but less than that of Bf-Gsx-Up1 + 2b (42.3 %) (Fig. 2a). However, the second construct, Bf-Gsx-Up1a-2b (−590 to −128 bp) had the same 3′ boundary as Gsx-Up1a, mirroring the split in Bf-Gsx-Up1a/Bf-Gsx-Up1b and breaking of the Up1c region seen earlier (Fig. 2a). Bf-Gsx-Up1a-2b, abolished both nerve cord and sensory vesicle expression, and in fact showed no CNS expression at all. Thus, the intact Gsx-Up1c region is absolutely required for CNS expression, whilst the conserved region extending more 5′ cannot drive expression on its own, but does boost the efficiency of the minimal Up1c region.

### TCF/Lef sites show unequal contribution to CNS expression domains

**Fig. 5:**
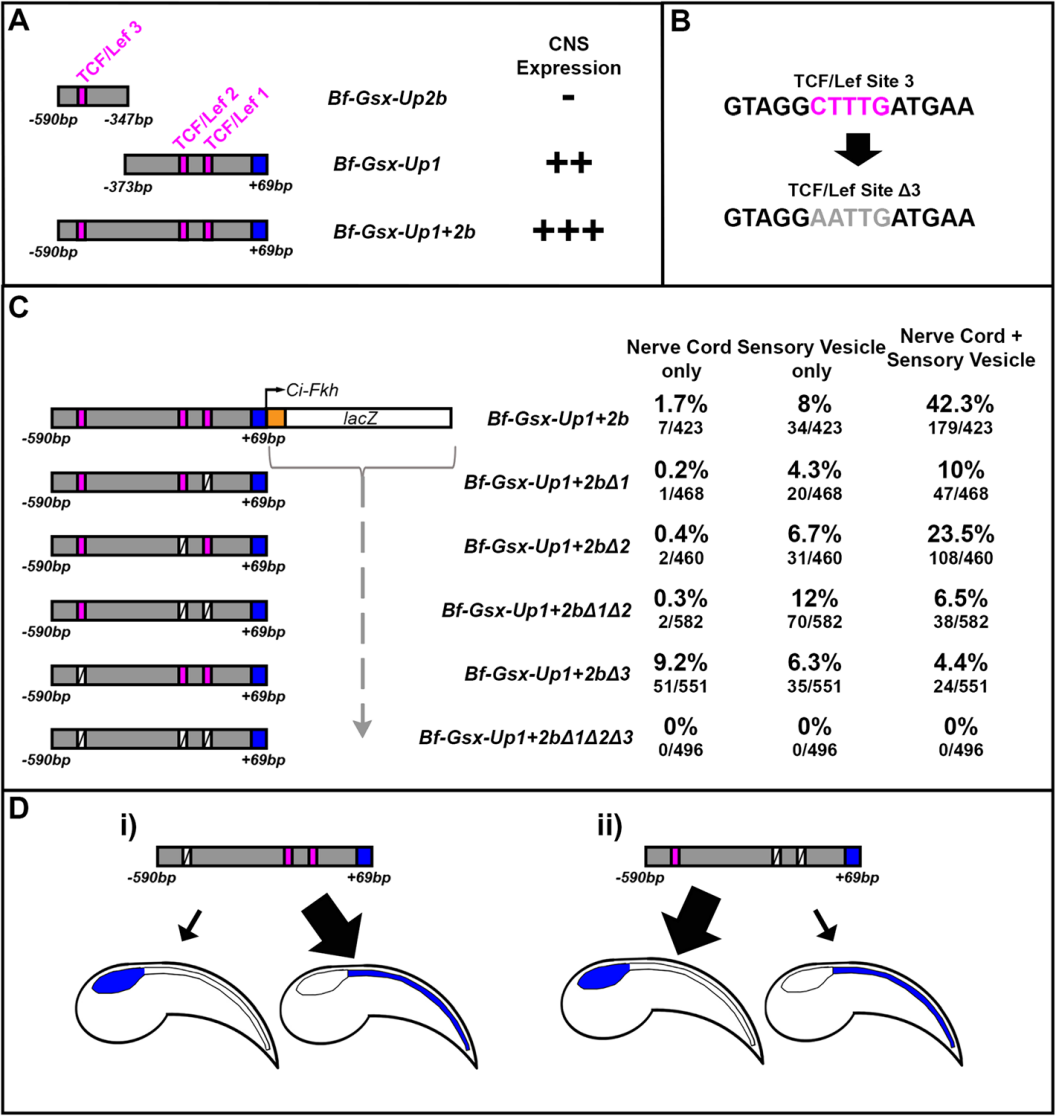
Mutation of TCF/Lef sites within the Bf-Gsx-Up1 + 2b construct. **a** Relative positions of TCF/Lef sites within the Bf-Gsx-Up2b, Bf-Gsx-Up1 and Bf-Gsx-Up1 + 2b regulatory regions. An additional TCF/Lef site within the 2b region is added in the Bf-Gsx-Up1 + 2b construct. ‘+’ symbols denote the presence of LacZ expression in the corresponding construct, with ‘++’ denoting high LacZ expression and ‘+++’ very high LacZ expression, whereas ‘–‘ denotes the absence of LacZ expression. (**b**) Schematic showing the DNA sequence of TCF/Lef site 3 before and after mutagenesis. Pink sequence denotes the TCF/Lef site ‘core’ sequence before mutation, whereas light gray sequence denotes the TCF/Lef site ‘core’ sequence after mutation. (**c**) Comparison of CNS expression incidence in the Bf-Gsx-Up1 + 2b construct with TCF/Lef binding motif mutants. The numbers of embryos displaying either nerve cord only, sensory vesicle only, or the nerve cord with sensory vesicle LacZ expression patterns have been recorded both as a percentage of the total number of embryos that developed and as raw numbers of embryos expressing LacZ for each domain alongside the total number of embryos that developed. Pink boxes denote the positions of intact TCF/Lef sites, whereas white crossed boxes indicate the positions of mutated TCF/Lef sites. (**d**) Schematic showing the partial division of function into ‘nerve cord’ and ‘sensory vesicle’ domains across the Bf-Gsx-Up1 + 2b regulatory element. (**d** *i*) Shows the bias of the 3' region and TCF/Lef sites 1 and 2 to drive nerve cord expression over sensory vesicle expression whilst (**d** *ii*) shows the bias of the 5' region and TCF/Lef site 3 to drive sensory vesicle expression over nerve cord expression

The role of TCF/Lef in the function of the Bf-Gsx-Up1 and Up1c regulatory regions prompted us to search for further sites that may be contributing towards the increase in expression efficiency seen in those constructs also containing the 2b region. As the Bf-Gsx-Up1/Up1c regions showed at least some collaborative effect between TCF/Lef sites, a third site located within the Gsx-Up2b region provided a good target for further mutagenesis (Fig. 5a). By analysing the effect of mutation on this third site, again as both a single mutation and in all possible permutations with the existing ‘core’ TCF/Lef Δ1 and Δ2 mutations, we aimed to determine if TCF/Lef site function was acting cumulatively and could account for the increase in expression seen in Bf-Gsx-Up1 + 2b and Up1c-2b, or if this third site could perhaps buffer against mutations in the ‘core’ Up1c region, providing a level of redundancy. Thus, the SiteΔ3 mutation, GTAGG**AATTG**ATGAA was produced (Fig. 5b). Bf-Gsx-Up1 + 2bΔ1, containing a mutation of the first ‘core’ TCF/Lef site, showed a dramatic decrease in CNS expression overall, though it is most apparent in the nerve cord with sensory vesicle expression, which decreases from 42.3 % in the wild type Bf-Gsx-Up1 + 2b to 10 % in the Bf-Gsx-Up1 + 2bΔ1 mutant. Interestingly, the Bf-Gsx-Up1 + 2bΔ2 mutated construct shows a less significant decrease in expression in the nerve cord with sensory vesicle expression pattern (23.5 %), though nerve cord alone (0.4 %) and sensory vesicle alone (6.7 %) both show comparable results to that of the Bf-Gsx-Up1 + 2bΔ1 construct (0.2 and 4.3 % respectively) (Fig. 5c). These results reveal a disparity in the contribution to regulatory function between site1 and site2, perhaps explained by the non-canonical binding sequence of site2, and the ability of site3 to compensate for this lower affinity site. However, if both site1 and site2 are mutated, as in the Bf-Gsx-Up1 + 2bΔ1Δ2 construct, we see that nerve cord with sensory vesicle expression decreases dramatically from 42.3 % in the WT to 6.5 % in the Δ1Δ2 mutation (Fig. 5c). This is also lower than either single core site mutation alone, supporting the idea that these TCF/Lef sites are functioning cumulatively. However, it is notable that there is not a complete abolition of expression as in the Bf-Gsx-Up1Δ1Δ2 and Bf-Gsx-Up1cΔ1Δ2 constructs (Figs. 4c, d and 5c), implying that the third site is able to partially compensate for the lack of TCF/Lef binding in the Core Up1c region.

The 3′ deletions of the Gsx-Up1 + 2b region, in Bf-Gsx-Up1a-2b, show that the Up1c region is required for CNS expression, even in the presence of a functional site3 binding site in the 2b region, suggesting that other transcription factors with binding sites in the Up1c core may be required to activate expression. Another particularly interesting observation from the Bf-Gsx-Up1 + 2bΔ1Δ2 construct is that sensory vesicle alone expression appears to be increased above that of the WT, from 8 to 12 % in the Δ1Δ2 mutation (Fig. 5c). This ‘increase’ in the sensory vesicle expression is indicative of a loss of nerve cord expression in embryos that would otherwise exhibit the nerve cord with sensory vesicle expression pattern, and that TCF/Lef site3 promotes a biased expression towards that of sensory vesicle rather than nerve cord. The converse mutation, with site3 mutated and the core site1 and site 2 intact, shows the opposite outcome to this. Bf-Gsx-Up1 + 2bΔ3 shows a decrease in both the nerve cord with sensory vesicle, and sensory vesicle alone phenotypes, but this time has ‘nerve cord only’ expression increased above that of the WT Bf-Gsx-Up1 + 2b region (from 1.7 % in the WT to 9.2 % in Δ3). This is likely representative of a loss of sensory vesicle expression from animals that would otherwise have the full nerve cord with sensory vesicle expression pattern. This leads to a model where the core Up1c TCF/Lef sites contribute more heavily, but not exclusively, to expression in nerve cord (Fig. 5d (i)) and TCF/Lef site 3, in the Gsx-Up2b region, contributes more heavily, but again not exclusively, to sensory vesicle expression (Fig. 5d (ii)). The final construct, with all three TCF/Lef site mutations, Bf-Gsx-Up1 + 2bΔ1Δ2Δ3 bolsters the previous evidence from Bf-Gsx-Up1Δ1Δ2 and Bf-Gsx-Up1c Δ1Δ2, showing that whilst other transcription factors may be involved in refining the output of this regulatory element, if all TCF/Lef binding sites are mutated then CNS expression is completely abolished and it is this transcription factor that provides the principal activation input (Fig. 4c, d and Fig. 5c).

### Mutation of TCF/Lef sites unmasks a latent repressive function

**Fig. 6:**
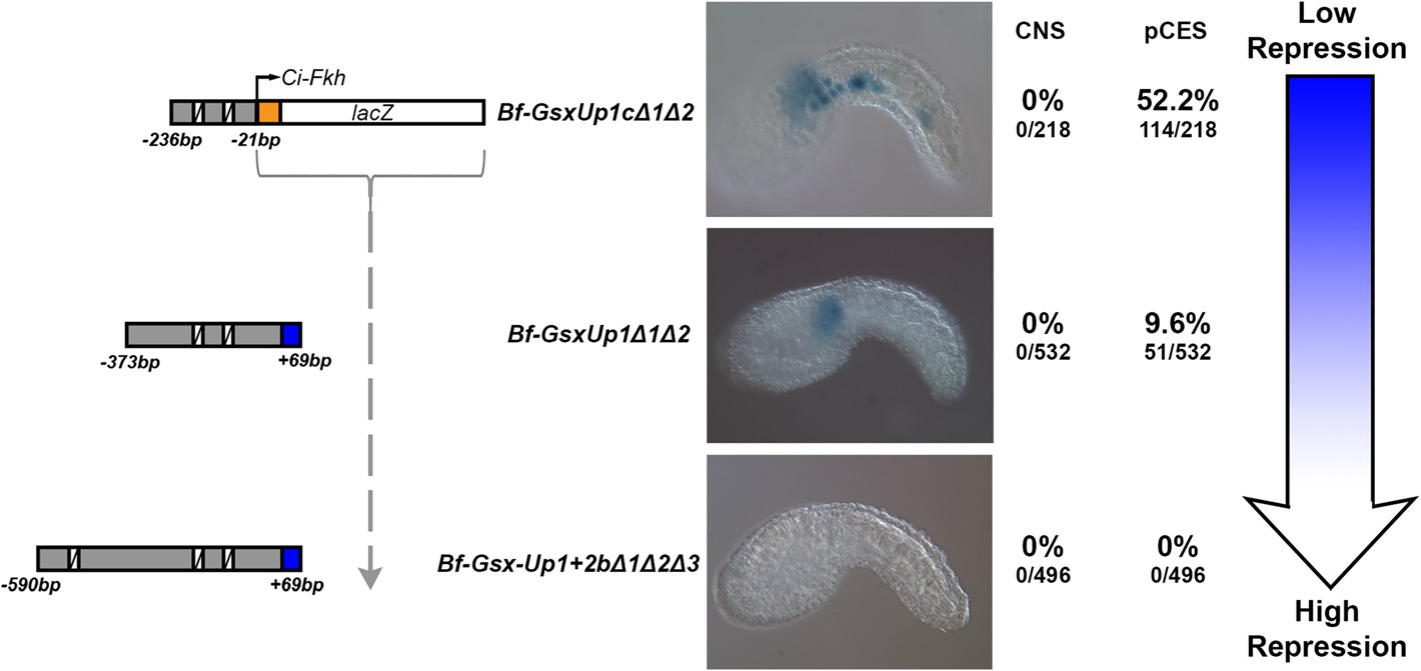
Mutation of all TCF/Lef sites abolishes CNS expression and reveals a latent repressive function that increases with regulatory element length. Images of mid tailbud *Ciona* embryos represent the maximal example of pCES ‘background’ LacZ expression for Bf-Gsx-Up1cΔ1Δ2, Bf-Gsx-Up1Δ1Δ2 and Bf-Gsx-Up1 + 2bΔ1Δ2Δ3 respectively. The numbers and percentage of embryos displaying either of the CNS LacZ expression domains examined in Figs. 2, 4 and 5 along with those displaying only pCES background expression. Numbers of embryos showing either pattern have been recorded both as a percentage of the total number of embryos that developed and as raw numbers of embryos expressing LacZ for each domain alongside the total number of embryos that developed. White crossed boxes indicate the positions of mutated TCF/Lef sites. The blue graduated arrow represents the decrease in pCES background expression associated with an increase in construct length from Bf-Gsx-Up1cΔ1Δ2 to Bf-Gsx-Up1Δ1Δ2, to Bf-Gsx-Up1 + 2bΔ1Δ2Δ3

When comparing expression in constructs with all TCF/Lef sites mutated, it was notable that the background head mesenchyme and tail muscle expression inherent to the pCES construct was different between Bf-Gsx-Up1cΔ1Δ2, Bf-Gsx-Up1Δ1Δ2 and Bf-Gsx-Up1 + 2bΔ1Δ2Δ3. As the TCF/Lef mutant constructs became longer, from Bf-Gsx-Up1cΔ1Δ2 as the smallest to Bf-Gsx-Up1 + 2bΔ1Δ2Δ3 as the longest, the pCES background expression also decreased. This led to, alongside the lack of CNS expression in all of these constructs, high pCES background in Bf-Gsx-Up1cΔ1Δ2 (52.2 % of embryos), a small amount of pCES background in Bf-Gsx-Up1Δ1Δ2 (9.6 % of embryos), and a complete abolition of any expression in the longer Bf-Gsx-Up1 + 2bΔ1Δ2Δ3 (0 % of embryos) (Fig. 6). Numbers of embryos displaying background pCES expression within the wild-type constructs are as follows; Bf-Gsx-Up1c, 88.2 % of embryos (255/289), Bf-Gsx-Up1, 45.5 % of embryos (156/343), and Bf-Gsx-Up1 + 2b, 10.9 % of embryos (46/423). This then shows a decrease in the levels of pCES background expression in direct response to TCF/Lef site mutation, with Bf-Gsx-Up1c > Bf-Gsx-Up1cΔ1Δ2 (decreasing from 88.2 to 52.2 %), Bf-Gsx-Up1 > Bf-Gsx-Up1Δ1Δ2 (decreasing from 45.5 to 9.6 %), and Bf-Gsx-Up1 + 2b > Bf-Gsx-Up1 + 2bΔ1Δ2Δ3 (decreasing from 10.9 to 0 %). It should be noted that pCES background expression levels in embryos containing the empty pCES vector (i.e. the reporter with no regulatory element insertion) lies at 94.6 % of embryos (142/150), whilst a long but non-functional region such as Bf-Gsx-Up2 (479 bp) has pCES background within 88.8 % of embryos (120/135). This suggests that the decrease in pCES background seen in response to increased construct length is also specific to the Bf-Gsx-Up1 + 2b region. We hypothesise that by removing TCF/Lef activation, a latent repressive function is unmasked that is spread throughout the regulatory region. Thus, in the absence of TCF/Lef binding, as the region inserted into the pCES multiple cloning site increases in size, it becomes more able to repress the background activity of the forkhead promoter. This system, where transcription factor binding is required to drive expression, but absence causes the regulatory element to actively repress gene expression, is one that could have widespread implications for gene regulation, allowing a regulatory region to have even greater precision over the control of the expression of its target gene, only allowing expression in the presence of a key, primary transcription factor, which in this case is TCF/Lef.

**Fig. 7:**
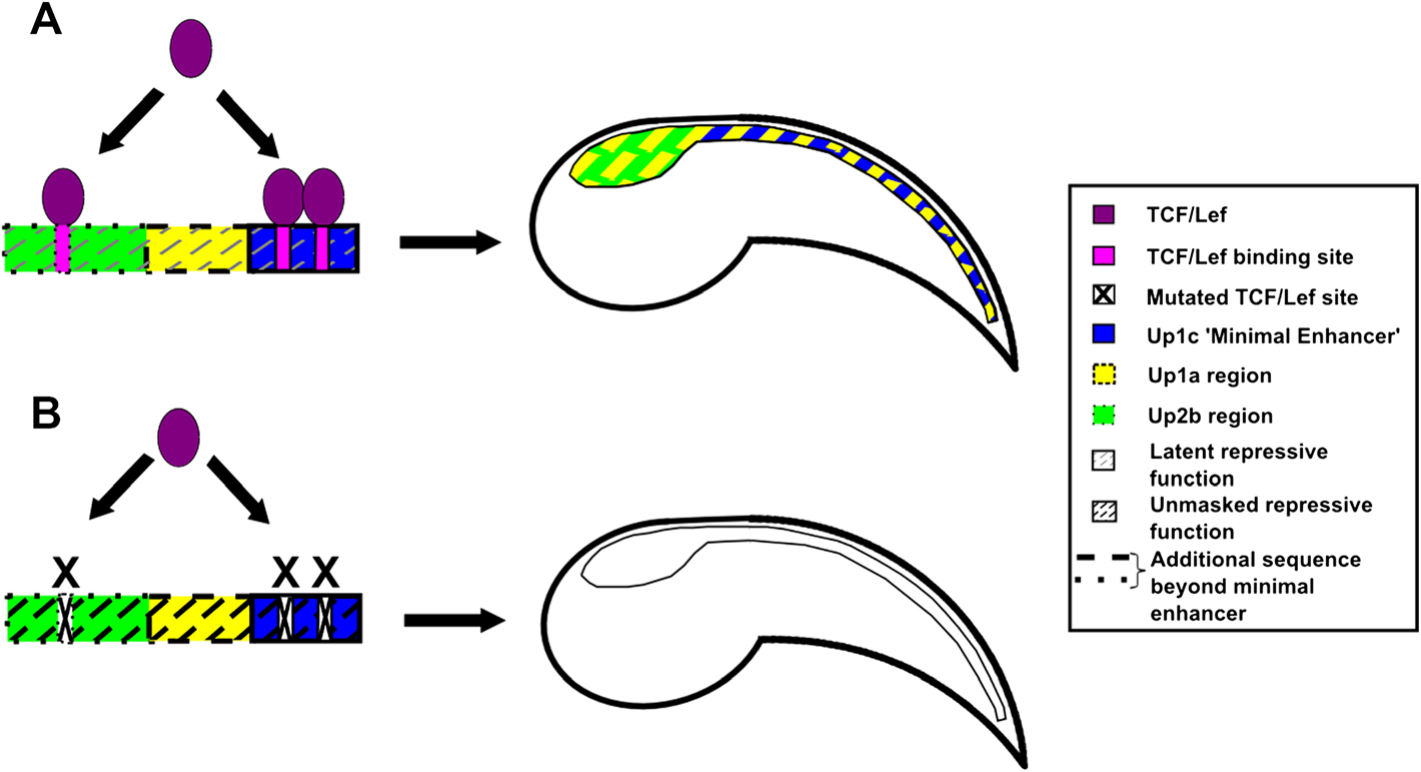
Model for the mode of action of the Bf-Gsx-Up1 + 2b regulatory element. **a** In the presence of TCF/Lef binding, CNS expression is activated. Additional sequence beyond the Up1c minimal enhancer both increases the efficiency of CNS expression and reveals a partial division of function into the Up1c minimal enhancer nerve cord domain and the Up2b region sensory vesicle domain. The intermediate Up1a region contributes partially to both expression domains. **b** In the absence of TCF/Lef binding, a latent repressive function is unmasked, preventing expression, even of the reporter vector background domains (not illustrated; see text for details).

## Discussion

### *C. intestinalis* electroporation provides an amenable system for examining chordate ParaHox regulation

This study has identified a regulatory region upstream of the amphioxus ParaHox gene Gsx that drives reporter gene expression in the CNS of *C. intestinalis. C. intestinalis* is currently a much more tractable system than amphioxus in which to perform reporter transgenics and rapidly characterise gene regulatory regions [64, 65]. The ability of an amphioxus *Gsx* regulatory element to drive strong, efficient and reproducible expression in *C. intestinalis* is thus promising as a system for examining ParaHox regulation via this cross-species transgenic approach. The conservation of ParaHox gene expression in the CNS allows us to dissect ParaHox regulation in *Ciona*, as the CNS of *Ciona* shows clear similarities in gene expression to the wider Chordata, perhaps even the Bilateria [66, 67]. In addition, the tunicates also retain many of the signalling pathways involved in Hox and ParaHox regulation, such as RA [68–70], FGF [71–73], BMP [74, 75], Wnts [76, 77] and hedgehog [76, 78, 79]. By using *C. intestinalis*, we are able to quickly assess hundreds of embryos at a time, allowing the identification of even weak regulatory elements, e.g. Bf-Gsx-Up1c. With this approach, even subtle differences in expression between different mutations are observed, allowing a more precise characterisation of regulatory function. This approach could also be applied more broadly across the ParaHox cluster, using the conservation of non-coding sequence between amphioxus species to inform functional studies. With the recent release of the Chinese amphioxus, *B. belcheri*, genome as well as the impending release of the European amphioxus, *B. lanceolatum*, genome and epigenomic data (personal communication with Dr Hector Escriva, CNRS, Observatoire Océanologique, Banyuls su Mer, France) we will be able to rapidly identify candidate regulatory regions, and carry out both initial screens and then more detailed characterisations of regulatory function using the *C. intestinalis* reporter system.

### The amphioxus Gsx-Up regulatory region recapitulates aspects of conserved chordate Gsx expression in the CNS

The ability of the Bf-Gsx-Up regulatory regions to drive LacZ expression throughout the CNS of *C. intestinalis* is intriguing, as these reporters show LacZ expression in homologous tissues to those expressing *Gsx* in amphioxus, i.e. the neural tube and the cerebral/sensory vesicle. The partial division of function observed in the Bf-Gsx-Up1 + 2b region into nerve cord and sensory vesicle domains may be linked to the two domains of native amphioxus expression. In this case, the visceral ganglion and nerve cord domain produced by the Bf-Gsx-Up region could correspond to the early domain of amphioxus *Gsx*, expressed at the level of somite 5 [4], whilst the observed sensory vesicle domain might then correspond to the later cerebral vesicle domain of amphioxus *Gsx*. The lack of expression in the most anterior sensory/cerebral vesicle region in both cases supports this and may indicate a defined boundary that is present in both the *C. intesinalis* and amphioxus anterior CNS.

Whilst there are obvious similarities between the Bf-Gsx-Up reporter expression in *C. intestinalis* and native amphioxus *Gsx* expression, it does remain more expansive within the *C. intestinalis* CNS than would be expected from amphioxus *Gsx* and endogenous *C. intestinalis Gsx* expression, specifically throughout the tail nerve cord. One explanation for this much broader reporter expression could be that the Bf-Gsx-Up regulatory element functions in conjunction with additional repressive elements that would otherwise spatiotemporally restrict expression. Such repressive elements would lie outside of the Bf-Gsx-Up region and so not lend function to the Bf-Gsx-Up reporters. Alternatively, it is possible that the repressive transcription factor system functioning to restrict expression in amphioxus does not exist in *C. intestinalis*, or is too divergent between the two species to provide function to the Bf-Gsx-Up reporter. Divergence of transcription factors and their binding sites is one obvious limitation of performing cross-species transgenesis and the future development of a more tractable and reliable reporter technique in amphioxus will help to overcome this limitation.

Within the wider chordate phylum, it is possible that there is some conserved regulation of *Gsx*, as there is the conservation of expression within the anterior CNS, particularly the ‘hindbrain’ and mid-forebrain as distinct domains of *Gsx* expression. In amphioxus, the early domain of *Gsx* expression is at the level of somite 5, a region thought to have homology to the vertebrate hindbrain [80, 81]. Also, for vertebrate Gsx genes, the hindbrain domain is also the first to be expressed, as seen in medaka [47], *Xenopus* [44], and mouse [45, 46]. This indicates that there is possibly a conserved regulatory program within the chordates that leads to the expression of this initial hindbrain domain. In addition, the expression of Gsx genes [44] within the mid-forebrain of vertebrates [46, 47], the cerebral vesicle of amphioxus [4], and the sensory vesicle of *C. intestinalis* [48] again hints at conserved regulation of Gsx within the chordates. This, in conjunction with the ability of the Bf-Gsx-Up reporter to drive expression throughout the CNS of *C. intestinalis*, suggests that a conserved regulatory pathway may be driving chordate Gsx expression.

### A role for TCF/Lef in Gsx regulation

The Bf-Gsx-Up regulatory regions show a clear and strong response to the mutation of TCF/Lef binding sites, requiring them to be intact in order to drive CNS expression in *C. intestinalis*. In concordance with this, native *C.intestinalis TCF/Lef* expression is consistent with a role for TCF/Lef in the direct regulation of Bf-Gsx-Up, as expression is present in the neural plate, then later in both the sensory vesicle and throughout the tail nerve cord and visceral ganglion, albeit weakly (Fig. 3) [79] (see ANISEED database [60]), which are all domains that the Bf-Gsx-Up regulatory regions drive expression in. In addition to these CNS domains, *C. intestinalis TCF/Lef* is also expressed in the head/neck mesenchyme where the background pCES expression remains high even in the presence of a strong enhancer such as Bf-Gsx-Up1 + 2b, suggesting that the regulation of the reporters by TCF/Lef is allowing this head/neck mesenchyme domain to persist.

Looking to amphioxus, there is strong expression of *TCF/Lef* within the cerebral vesicle, correlating with stages where *Gsx* is expressed in the same tissue [82]. This raises the distinct possibility that the Bf-Gsx-Up region responds to the same signal in its native environment. Though no strong *TCF/Lef* domain has yet been observed in the position of the early amphioxus *Gsx* domain [4], it is possible that weak *TCF/Lef* expression present in the nerve cord of amphioxus may be sufficient to allow *Gsx* expression, with other transcription factors acting to restrict the expression domain.

In the vertebrates, the expression of TCF/Lef family members are similar to that of *C. intestinalis TCF/Lef*, with strong expression present throughout the neural tube [83]. The presence of TCF/Lef expression in the CNS of all three chordate sub-phyla shows a conservation of expression within these tissues, which along with the association of *TCF/Lef* with amphioxus *Gsx* regulation in the Bf-Gsx-Up reporters, implies an ancestral role for TCF/Lef in the direct regulation of chordate Gsx. In order to confirm this hypothesis, future work would be required to establish whether there is similar direct regulation of *C. intestinalis* and vertebrate Gsx genes by TCF/Lef.

### Unravelling complex cis-regulatory function and multiple levels of regulation

Within the Bf-Gsx-Up1 + 2b regulatory element, there is a differential response to the mutation of TCF/Lef sites across the regulatory region. Sites within the 3′ minimal Up1c region contribute with a bias toward nerve cord expression whereas the third site, within the 5′ Up2b region, contributes with a bias toward sensory vesicle expression (Fig. 5d). The presence of a single intact TCF/Lef site still allows for expression of the whole CNS expression pattern even with this bias present however (Fig. 5c), suggesting that TCF/Lef is required to activate expression, but not necessarily specify more restricted expression domains. In addition, the ability of a single TCF/Lef site to allow the whole CNS expression pattern implies that TCF/Lef binding in one area permits the activity of other factors elsewhere in the regulatory region, implying cross-talk between factors on binding sites in different regions of the Bf-Gsx-Up regulatory element. Further work would be required to identify the factors involved in this CNS regionalisation, looking at the transcription factors expressed within the CNS of both *C. intestinalis* and amphioxus.

The ability of our Bf-Gsx-Up regulatory region to not only drive expression in the presence of intact TCF/Lef sites, but also to actively repress expression if these sites do not remain intact, demonstrates another layer of control within this regulatory region (summary model in Fig. 7). In addition to the partial division of regulatory function into different domains, once TCF/Lef binding is abolished a latent repressive function is unmasked as, currently unknown, repressive factors are able to silence gene expression, preventing any background ectopic pCES transcription in the absence of TCF/Lef (Fig. 6). This repressive state may even be the ‘default’ for Bf-Gsx-Up, which would then switch from repressor to enhancer in the presence of TCF/Lef binding. Characterization of the nature of this repressor activity will be an interesting avenue for future research.

### Building a picture of ancestral ParaHox regulation

TCF/Lef is likely to be directly regulating amphioxus *Gsx*, and though further research is needed to confirm TCF/Lef binding, this is the first evidence of such an interaction with Gsx genes. If amphioxus *Gsx* is directly regulated by TCF/Lef, this now provides examples of TCF/Lef regulation across all three ParaHox genes within the chordates. *Xenopus Xlhbox8* (*Xlox*) has been shown to be induced by mouse *TCF4* and human *Lef-1* in animal cap explants, where it normally plays a role in the specification of the duodenum and pancreatic tissues [30]. In pancreatic islet cell culture, *TCF7L2* has also been shown to regulate *PDX1* (Xlox) [31]. The posterior ParaHox gene, Cdx, also responds to TCF/Lef, and work on mouse *Cdx1* promoters shows that TCF/Lef- β-catenin complex binding is required to activate *Cdx1* expression and embryos suffer abrogated *Cdx1* expression in the small intestine in response to *TCF4* null mutants [9]. These data, in addition to our own, suggests that all three ParaHox genes may have been ancestrally regulated by TCF/Lef. Indeed, TCF/Lef expression patterns in vertebrates as well as amphioxus show strong expression in both the CNS and in the gut [30, 82–84]. The presence of amphioxus *TCF/Lef* in this posterior gut region [82] makes experiments to determine if amphioxus *Xlox* and *Cdx* are also regulated by this transcription factor an interesting prospect.

Whilst evidence for direct TCF/Lef involvement in the regulation of ParaHox genes is currently limited to the chordates, data suggests that Wnt signalling may play a wider role in the regulation of Xlox and Cdx within the Bilateria. This holds particular relevance as TCF/Lef family members have been shown to act in a complex with β-catenin as the downstream transcriptional activator of canonical Wnt signalling during embryogenesis [85, 86]. This pathway has also been shown to be crucial to the proper development of the gut and neural tube [87–89], and has been well studied as a regulator of vertebrate Cdx genes in these tissues [9, 23–25, 28, 90]. This may even have been present at the base of the chordates, as Wnt may also be regulating amphioxus *Cdx*. [91] treated amphioxus embryos with Li^+^, to upregulate Wnt/β-catenin signalling via inhibition of GSK3β, which resulted in an ectopic anterior domain of *AmphiCdx* as well as reduction of the CNS domain and expansion in the hindgut. Cdx has also been shown to respond to Wnt signalling in the growth zone of both the beetle *Tribolium castaneum* [35] and the spider *Parasteatoda tepidariorum* [92], suggesting that the regulation of Cdx by Wnt signalling may also have been present at the base of the arthropods [93], and perhaps Bilateria, and is in concordance with the prevalence of Wnt signalling as a posterior axial patterning signal across many phyla (reviewed in [94]).

The origin of Xlox regulation by Wnt-TCF/Lef is much harder to determine, as Xlox appears to have been lost from the ecdysozoans sampled so far (with the possible exception of *Strigamia maritima* [95]), but studies of the Xlox/Cdx boundary in the gut of deuterostomes may yield insights. This Xlox/Cdx boundary is a feature that is highly conserved in the deuterostomes, present not only in the chordates but also the echinoderms [96]. In the sea urchin *Strongylocentrotus purpuratus*, Wnt signalling has been implicated in the formation of the Xlox/Cdx gut boundary where it influences the expression of Xlox [10], suggesting a conserved regulatory mechanism at the base of the deuterostomes. It is also possible that Wnt may interact with RA to determine this boundary, as altered RA signalling has been shown to alter the position of this Xlox/Cdx boundary in amphioxus [4], and *Cdx1* in vertebrates is known to respond directly to combinatorial inputs from both the RA and Wnt signalling pathways [26].

Little work has been done on the regulation of Gsx but gene expression within the brain of *Drosophila melanogaster* suggests that Wnt signalling may also be playing a role in the regulation of Gsx, with *Wingless* (Wg) active within *Intermediate neuroblasts defective* (*Ind*) (Gsx)-positive brain neuroblasts. It is also possible that Wnt signalling could be functioning upstream of the Bf-Gsx-Up regulatory region, acting via the TCF/Lef-mediated canonical Wnt pathway, as Wnt ligands are expressed in the neural tube of both *C. intestinalis* [79] and amphioxus [97, 98], though further work would be needed to determine if Wnt really is coordinating TCF/Lef binding in the Bf-Gsx-Up regulatory region. If these are cases of direct regulation of Gsx by Wnt signalling, it is possible that a Wnt-Gsx regulatory pathway was present at the base of the Bilateria. Wnt signalling, via TCF/Lef, may even function as a pan-cluster regulatory mechanism within the ParaHox cluster, acting upon all three ParaHox genes, and further regulatory studies across distant taxa and multiple ParaHox genes would help to elucidate this Wnt-TCF/Lef ParaHox regulatory hypothesis.

Looking even further back into the evolution of animals, the involvement of Wnt signalling in anterior-posterior patterning also appears to play a role within the Cnidaria [99, 100] and even sponges [101–105]. Given that the ParaHox genes are now thought to have originated in the last common ancestor of animals [18, 106, 107] and the Wnt system is similarly ancient, then there may well have been a direct ParaHox-TCF/Lef link from the earliest stages of animal evolution.

## Conclusions

In this study, a cross species transgenic approach is used to examine the regulatory mechanisms controlling the Gsx ParaHox gene in a representative of the basal lineage of the chordates, by using *Ciona* as a ‘living test tube’ in which to dissect amphioxus regulatory elements. We have identified and characterised a regulatory element upstream of *B. floridae Gsx* that drives expression of a LacZ reporter construct within the CNS of transgenic *C. intestinalis* embryos. Deletion analysis has revealed a minimal enhancer region, Bf-Gsx-Up1c, which is both sufficient and required to drive reporter expression within the *Ciona* CNS. Sequence comparison both between *B. floridae* individuals and across amphioxus species allowed the identification of the larger, conserved and much more efficient Bf-Gsx1 + 2b region. TCF/Lef binding sites were found to be required for CNS expression of Bf-Gsx-Up constructs and mutation of TCF/Lef binding sites resulted not only in the loss of CNS expression, but also revealed the unequal contribution of TCF/Lef binding sites to the sensory vesicle and nerve cord expression domains. Finally, in the absence of intact TCF/Lef binding sites, a latent repressive function is unmasked. The conserved expression of both Gsx and TCF/Lef within the CNS of chordates suggests the ancestral regulation of Gsx by TCF/Lef at the base of the chordates. Furthermore, evidence suggests that all three ParaHox genes may be regulated by TCF/Lef within the chordates, and the importance of TCF/Lef and Wnt signalling in A/P patterning raises the possibility of TCF/Lef as a deeply conserved regulatory factor directing ParaHox expression across the Bilateria, or even Metazoa.

## Methods

### Cloning of *B. floridae Gsx* upstream regulatory elements

A 2.1Kb upstream region of *B. floridae Gsx* was obtained by PCR from PAC clone 33B4 [108]. PCR was carried out using the High fidelity Pwo-polymerase (Peqlab). Purified products were then A-tailed using a 15 min Taq-polymerase reaction at 72 °C. Primer sequences and annealing temperatures used are provided in Additional file 3: Table S1. The 1.7 kb Bf-Gsx-Up-Proximal regulatory region (Fig. 1a) was then obtained by further PCR from this upstream region, with subsequent smaller regions obtained from this 1.7 kb Bf-Gsx-Up region. PCR reactions were all carried out using the following PCR program. A 2 min denaturation at 94 °C followed by 10 cycles of; 15 s at 94 °C, 30s annealing (see Additional file 3: Table S1 for annealing temperature), 45 s/Kb elongation at 72 °C, which was followed by 20 cycles of; 15 s at 94 °C, 30s annealing, 45 s/Kb elongation + 5 s/cycle at 72 °C and then ended with a final elongation of 4 min at 72 °C. Primers for Bf-Gsx-Up-Proximal and smaller regions contained a 5′ PstI site on the forward primer and 3′ BamHI site on the reverse primer. These restriction sites facilitated directional cloning into the multiple cloning site of the pCES expression vector (kindly gifted by Dr Clare Hudson, CNRS, Villefranche sur Mer, France) [64] with minimal flanking sequence, after shuttling through pGEM-T Easy (Promega). pCES contains a multiple cloning site upstream of a forkhead promoter coupled to a LacZ reporter gene.

### Animal husbandry

Wild *C. intestinalis* were collected from two different sites; Croabh Haven, Scotland during May to July and from Arbroath, Scotland in August and September. The differing seawater temperatures at the two sites allowed us to extend the season in which embryos could be collected. Animals were collected from pontoons located in a marina (Croabh Haven) or a small harbour (Arbroath) and then maintained in a flow-through aquarium system with seawater pumped in directly from the North Sea, filtered and pumped into 50 L tanks with aeration. Water was allowed to drain out of the tanks and replenished at a constant rate with fresh seawater, keeping a steady flow across the tank. Animals were kept submerged within plastic baskets held up by polystyrene floats, to allow waste to fall to the bottom of the tank rather than it accumulating around the animals. Some food entered as a constant flow of algae provided from the seawater inflow, and this was supplemented once a day with a mixed suspension of *Rhinomonas reticulata* var. *reticulata* (strain number CCAP 995/2), a unicellular red alga, supplied by the Scottish Association for Marine Science (SAMS, Oban, Scotland), and *Tetraselmis sp*., a unicellular lipid-rich green alga, supplied by Florida Aqua Farms (Dade City, Florida, United States). These algae were grown in culture and concentrated by low-speed centrifugation before 5 ml of mixed, concentrated culture was added to the tank by pipette. All in-flow and out-flow of seawater was stopped for 2 h whilst the animals were fed the algal mix. The presence of a dark green colouration in the gut was used as a visual cue to indicate successful feeding. In order to collect gametes, gravid animals, as distinguished by an abundance of pink/orange eggs visible through the body wall, were selected and gametes liberated by dissection.

### Electroporation and *C. intestinalis* transgenes

Electroporation was carried out using a custom-built electroporator based on the details provided by Zeller et al. [109]. This was used with settings at 50 V, 1000 F and either 30 or 40 Ω. *C. intestinalis* transgenics were produced by electroporation of fertilised eggs according to Corbo et al. [53], with the following modifications. All microfuge tubes, 15 ml tubes and Pasteur pipettes used in this protocol were silicon-coated using Sigmacote-SL2 (Sigma) to avoid embryos sticking to equipment. 40-50 μg of plasmid DNA was dissolved in 500 μl of 0.77 M D-Mannitol. Fertilised eggs were dechorionated using 2 % sodium thioglycolate and 0.1 % protease, prepared separately and then mixed prior to dechorionation. Dechorionation times varied for animals from different locations, with embryos from Croabh Haven requiring 3–3.5 min and embryos from Arbroath 6-8mins. These were then washed with filtered seawater several times, gently spinning via a hand centrifuge for no more than 2 min in between washes. No more than 200 μl of embryos were added to a microfuge tube and the 500 μl DNA/Mannitol mix added. This was mixed by gently pipetting and added to a microcuvette (BioRad, electrode width = 0.4 cm). After the pulse, the DNA/eggs were immediately transferred to a seawater/agarose-coated plate flooded with filtered sea water and 5 ml of 100 mg/ml Gentamycin. Embryos were then reared at 16 °C until the desired time point. All constructs were tested in triplicate in separate electroporations, with positive controls (known active constructs) used as well as pCES lacking any amphioxus DNA as a negative control.

### Identification of TCF/Lef sites

The Bf-Gsx-Up-Proximal regions of 7 different *B. floridae* individuals were isolated, sequenced and aligned with the corresponding regions taken from the PAC clone 33B4 and from the *B. floridae* genome (accessed at JGI, *B.floridae* Scaffold_116 http://genome.jgi.doe.gov/Brafl1/Brafl1.home.html). These sequences have been deposited in Genbank, with the accession numbers provided below. This alignment was carried out and viewed using VISTA [63, 110] to view the level of conservation across all sequences (Additional file 2: Figure S2). The highly conserved region was then processed using the MULAN multiTF program [59] in order to obtain a list of 87 potential transcription factor binding sites conserved in the Bf-Gsx-Up1 region of all individuals. This was then reduced by cross-referencing with the ANISEED database [60] for genes expressed in the neural plate of *C. intestinalis* to a total of 16. This was then further reduced to a set of 7 transcription factors that were expressed throughout the whole neural tube in a manner similar to the Bf-Gsx-Up1 construct. TCF/Lef was then focused on as all other factors were also expressed in tissues that did not express the Bf-Gsx-Up1 construct. In addition, the Bf-Gsx-Up-Proximal region was then cross-checked specifically for TCF/Lef sites to confirm their presence, using PROMO [111, 112] with a maximum dissimilarity rate of 15 %.

### Site directed mutagenesis

Site directed mutagenesis (SDM) on TCF/Lef sites was carried out using the Phusion SDM kit (Thermo Scientific) to introduce double point mutations. SDM was carried out using RP-HPLC purified 32 bp primers with 5′ phosphorylation modifications (Additional file 3: Table S1) according to the manufacturer’s instructions. SDM was performed on the pGEM-T-easy copy of each regulatory region, with inserts dropped out via restriction digest and then ligated into pCES.

### Cloning and In situ hybridisation of *Ci-TCF*

*C. intestinalis TCF/Lef* was cloned from *Ciona* cDNA. Total RNA was extracted from mixed mid-gastrula to late-tailbud stage embryos and extracted using the Isolate RNA mini kit (Bioline) according to the manufacturer’s instructions. An additional DNase I treatment was included during the protocol during the tissue lysis stage in order to fully remove genomic contamination, with 1 μl of DNase I (Fermentas) added to Lysis buffer R and incubated at 37 °C for 30 mins. This was then heat-deactivated at 65 °C for 10mins before being processed with the Isolate RNA mini kit (Bioline). cDNA was produced using the Tetro cDNA synthesis kit (Bioline). 22 bp primers were used (listed in Additional file 3: Table S1) to amplify a 641 bp segment covering part of the 5′ end of the *Ci-TCF/Lef* cDNA. The accession number for this sequence is provided below. This was cloned into pGEM-T-easy and a PCR template synthesised using M13 primers. An antisense RNA probe was then synthesised from this template using T7 polymerase. In situ hybridisation was carried out as detailed in [113] with the following modifications. Embryos were rehydrated through an ethanol series into PBT and then digested for 10 min at room temperature in 2 μg/ml proteinase K for gastrula to mid-tailbud embryos and 20 min for late-tailbud embryos. 4 μl of 10 % glycine was then added, swirled and the solution removed immediately and replaced with 2 mg/ml glycine in PBT and washed for 5 min. This was then changed for 4 % PFA in PBS and fixed for 1 h at room temperature. After triethanolamine/acetic anhydride washes, embryos were washed three times in PBT before being washed once in 50:50 Hybridisation buffer (HYB)(50 % Formamide, 5X SSC pH4.5, 100 μg/ml yeast RNA, 50 μg/ml heparin, 0.1 % Tween 20) to PBT, then once in HYB. This was then changed to fresh HYB and embryos were pre-hybridised at 60 °C for 3 h. Approximately 50–100 ng of probe in fresh HYB was denatured at 70 °C for 10 min before being added to the embryos. Embryos were then incubated at 70 °C for 2 min before being moved to an overnight hybridisation at 60 °C, rocking gently. Hybridised embryos then underwent 3x 20 min washes in *Ciona* Wash buffer 1 (50 % (v/v) Formamide, 5x SSC, 1 % (v/v) SDS) at hybridisation temperature. This was followed by 2x 20 min washes at 37 °C in *Ciona* wash buffer 2 (50 % (v/v) Formamide, 2x SSC, 1 % (v/v) SDS) and then 2x 5 min washes at room temperature in *Ciona* Wash buffer 3 (2x SSC, 0.1 % (v/v) Tween 20). 3x 5 min washes at 37 °C were followed by 2x 20 min washes at 50 °C, all in *Ciona* Wash buffer 3. A single wash for 20 min at 50 °C in *Ciona* wash buffer 4 (0.2x SSC, 0.1 % (v/v) Tween 20) was carried out before 3x 10 min washes in PBT at room temperature. Embryos were then blocked for 3 h in blocking solution (20 % heat treated sheep serum in 1x PBS, 0.1 % (v/v) Tween 20, 2 mg/ml BSA) before adding 1:2000 Anti-digoxigenin-AP Fab fragments in blocking solution and incubating O/N at 4 °C. No modifications were made to the staining and post-staining procedures.

### Accession numbers

Genbank accession numbers for sequences used in this study are as follows:

*B.floridae* Gsx upstream polymorphism: [Bf2_Gsx_Upstream: **KP739759**, Bf4_Gsx_Upstream: **KP739757**, Bf5_Gsx_Upstream: **KP739758**, Bf7_5_Gsx_Upstream: **KP739762**, Bf7_6_Gsx_Upstream: **KP739761**, Bf8_Gsx_Upstream: **KP739763**, Bf9_Gsx_Upstream: **KP739764**, Bf10_Gsx_Upstream: **KP739760**].

*C. intestinalis* TCF/Lef 5′ end (used for in-situ probe): [Ci_TCF-Lef_mRNA_641bp: **KP739765**]

## Additional files

Additional file 1: Figure S1. Ectopic expression of pCES vector in *Ciona intestinalis*. Expression is almost always seen in mesenchymal tissue (A-H) (arrows). Expression is activated during gastrulation stages in mesenchymal cell lineages (A). At later stages, expression is visible posterior to the mesenchyme in variable numbers of tail muscle cells (C-H). Expression is also, though very rarely, observed in the centre of the sensory vesicle (black arrowheads) of tailbud stage embryos (A-C) show dorsal views, whilst (D-H) show lateral views. Lower case lettering refers to the stage of development; g, gastrula; n, neurula; itb, initial tailbud; mtb, mid tailbud; ltb, late tailbud. Scale bars represent 100 μm. (PDF 2350 kb) Additional file 2: Figure S2. VISTA analysis of the polymorphic *AmphiGsx*-Upstream Proximal region. Sequences from multiple *Branchiostoma floridae* individuals, as well as from *Branchiostoma belcheri* were compared to the amphioxus ParaHox PAC sequence. The regions corresponding to the deletion constructs *Gsx*-Up3 and the 5′ half of *Gsx*-Up2 are the most variable, while the region covering *Gsx*-Up1 and the 3′ half of *Gsx*-Up2 is the most conserved. Note that Bf7_5 and Bf7_6 are different haplotypes from the same individual. Accession numbers for these sequences are found in the Methods. (PDF 1037 kb) Additional file 3: Table S1. Oligonucleotides used for cloning and mutagenesis. (DOCX 19 kb)

## References

[1] Ferrier DEK, Holland PWH (2001). Ancient origin of the Hox gene cluster. Nat Rev Genet.

[2] Mallo M, Alonso CR (2013). The regulation of Hox gene expression during animal development. Development.

[3] Ferrier DEK (2012). Evolution of the Hox gene cluster.

[4] Osborne PW, Benoit G, Laudet V, Schubert M, Ferrier DEK (2009). Differential regulation of ParaHox genes by retinoic acid in the invertebrate chordate amphioxus (*Branchiostoma floridae*). Dev Biol.

[5] Simeone A, Acampora D, Arcioni L, Andrews PW, Boncinelli E, Mavilio F (1990). Sequential activation of Hox2 homeobox genes by retinoic acid in human embryonal carcinoma-cells. Nature.

[6] Chambeyron S, Bickmore WA (2004). Chromatin decondensation and nuclear reorganization of the HoxB locus upon induction of transcription. Genes Dev.

[7] Maloof JN, Whangbo J, Harris JM, Jongeward GD, Kenyon C (1999). A Wnt signaling pathway controls Hox gene expression and neuroblast migration in *C. elegans*. Development.

[8] Rieden P, Vilaspasa FL, Durston AJ (2010). Xwnt8 directly initiates expression of *labial* Hox genes. Dev Dyn.

[9] Lickert H, Domon C, Huls G, Wehrle C, Duluc I, Clevers H, Meyer BI, Freund JN, Kemler R (2000). Wnt/beta-catenin signaling regulates the expression of the homeobox gene *Cdx1* in embryonic intestine. Development.

[10] Annunziata R, Arnone MI (2014). A dynamic regulatory network explains ParaHox gene control of gut patterning in the sea urchin. Development.

[11] Ferrier DEK, Minguillon C (2003). Evolution of the Hox/ParaHox gene clusters. Int J Dev Biol.

[12] Monteiro AS, Ferrier DEK (2006). Hox genes are not always colinear. Int J Biol Sci.

[13] Mulley JF, Chiu CH, Holland PWH (2006). Breakup of a homeobox cluster after genome duplication in teleosts. Proc Natl Acad Sci U S A.

[14] Hui JHL, Raible F, Korchagina N, Dray N, Samain S, Magdelenat G, Jubin C, Segurens B, Balavoine G, Arendt D (2009). Features of the ancestral bilaterian inferred from *Platynereis dumerilii* ParaHox genes. BMC Biol.

[15] Annunziata R, Martinez P, Arnone MI (2013). Intact cluster and chordate-like expression of ParaHox genes in a sea star. BMC Biol.

[16] Ikuta T, Chen Y-C, Annunziata R, Ting H-C, Tung C-H, Koyanagi R, Tagawa K, Humphreys T, Fujiyama A, Saiga H (2013). Identification of an intact ParaHox cluster with temporal colinearity but altered spatial colinearity in the hemichordate *Ptychodera flava*. BMC Evol Biol.

[17] Cañestro C, Postlethwait JH (2007). Development of a chordate anterior-posterior axis without classical retinoic acid signaling. Dev Biol.

[18] Garstang M, Ferrier DEK (2013). Time is of the essence for ParaHox homeobox gene clustering. BMC Biol.

[19] Kikuta H, Laplante M, Navratilova P, Komisarczuk AZ, Engstrom PG, Fredman D, Akalin A, Caccamo M, Sealy I, Howe K (2007). Genomic regulatory blocks encompass multiple neighboring genes and maintain conserved synteny in vertebrates. Genome Res.

[20] Lee AP, Koh EGL, Tay A, Brenner S, Venkatesh B (2006). Highly conserved syntenic blocks at the vertebrate Hox loci and conserved regulatory elements within and outside Hox gene clusters. Proc Natl Acad Sci U S A.

[21] Houle M, Sylvestre JR, Lohnes D (2003). Retinoic acid regulates a subset of *Cdx1* function in vivo. Development.

[22] Houle M, Prinos P, Iulianella A, Bouchard N, Lohnes D (2000). Retinoic acid regulation of *Cdx1*: an indirect mechanism for retinoids and vertebral specification. Mol Cell Biol.

[23] Lickert H, Kemler R (2002). Functional analysis of cis-regulatory elements controlling initiation and maintenance of early *Cdx1* gene expression in the mouse. Dev Dyn.

[24] Pilon N, Oh K, Sylvestre JR, Bouchard N, Savory J, Lohnes D (2006). *Cdx4* is a direct target of the canonical Wnt pathway. Dev Biol.

[25] Ikeya M, Takada S (2001). *Wnt-3a* is required for somite specification along the anteroposterior axis of the mouse embryo and for regulation of *cdx-1* expression. Mech Dev.

[26] Pilon N, Oh K, Sylvestre JR, Savory JGA, Lohnes D (2007). Wnt signaling is a key mediator of *Cdx1* expression in vivo. Development.

[27] Gaunt SJ, Paul YL (2014). Synergistic action in P19 pluripotential cells of retinoic acid and Wnt3a on *Cdx1* enhancer elements. Int J Dev Biol.

[28] Shimizu T, Bae YK, Muraoka O, Hibi M (2005). Interaction of Wnt and caudal-related genes in zebrafish posterior body formation. Dev Biol.

[29] Mazzoni EO, Mahony S, Peljto M, Patel T, Thornton SR, McCuine S, Reeder C, Boyer LA, Young RA, Gifford DK (2013). Saltatory remodeling of Hox chromatin in response to rostrocaudal patterning signals. Nat Neurosci.

[30] Lee YJ, Swencki B, Shoichet S, Shivdasani RA (1999). A possible role for the high mobility group box transcription factor *Tcf-4* in vertebrate gut epithelial cell differentiation. J Biol Chem.

[31] Zhou Y, Park S-Y, Su J, Bailey K, Ottosson-Laakso E, Shcherbina L, Oskolkov N, Zhang E, Thevenin T, Fadista J (2014). TCF7L2 is a master regulator of insulin production and processing. Hum Mol Genet.

[32] Stafford D, Prince VE (2002). Retinoic acid signaling is required for a critical early step in zebrafish pancreatic development. Curr Biol.

[33] Martin M, Gallego-Llamas J, Ribes V, Kedinger M, Niederreither K, Chambon P, Dolle P, Gradwohl G (2005). Dorsal pancreas agenesis in retinoic acid-deficient *Raldh2* mutant mice. Dev Biol.

[34] Molotkov A, Molotkova N, Duester G (2005). Retinoic acid generated by Raldh2 in mesoderm is required for mouse dorsal endodermal pancreas development. Dev Dyn.

[35] Oberhofer G, Grossmann D, Siemanowski JL, Beissbarth T, Bucher G (2014). Wnt/beta-catenin signaling integrates patterning and metabolism of the insect growth zone. Development.

[36] Cowden J, Levine M (2003). Ventral dominance governs sequential patterns of gene expression across the dorsal-ventral axis of the neuroectoderm in the *Drosophila* embryo. Dev Biol.

[37] Weiss JB, Von Ohlen T, Mellerick DM, Dressler G, Doe CQ, Scott MP (1998). Dorsoventral patterning in the *Drosophila* central nervous system: the *intermediate neuroblasts defective* homeobox gene specifies intermediate column identity. Genes Dev.

[38] Winterbottom EF, Illes JC, Faas L, Isaacs HV (2010). Conserved and novel roles for the Gsh2 transcription factor in primary neurogenesis. Development.

[39] Denes AS, Jekely G, Steinmetz PRH, Raible F, Snyman H, Prud’homme B, Ferrier DEK, Balavoine G, Arendt D (2007). Molecular architecture of annelid nerve cord supports common origin of nervous system centralization in Bilateria. Cell.

[40] Wheeler SR, Carrico ML, Wilson BA, Skeath JB (2005). The *Tribolium* columnar genes reveal conservation and plasticity in neural precursor patterning along the embryonic dorsal-ventral axis. Dev Biol.

[41] Samadi L, Steiner G (2010). Conservation of ParaHox genes’ function in patterning of the digestive tract of the marine gastropod *Gibbula varia*. BMC Dev Biol.

[42] Wollesen T, Rodríguez Monje SV, McDougall C, Degnan BM, Wanninger A (2015). The ParaHox gene *Gsx* patterns the apical organ and central nervous system but not the foregut in scaphopod and cephalopod mollusks. Evodevo.

[43] Cheesman SE, Eisen JS (2004). *Gsh1* demarcates hypothalamus and intermediate spinal cord in zebrafish. Gene Expr Patterns.

[44] Illes JC, Winterbottom E, Isaacs HV (2009). Cloning and expression analysis of the anterior ParaHox genes, *Gsh1* and *Gsh2* from *Xenopus tropicalis*. Dev Dyn.

[45] Valerius MT, Li H, Stock JL, Weinstein M, Kaur S, Singh G, Potter SS (1995). *Gsh-1* - A novel murine homeobox gene expressed in the central-nervous-system. Dev Dyn.

[46] Hsiehli HM, Witte DP, Szucsik JC, Weinstein M, Li H, Potter SS (1995). *Gsh-2*, a murine homeobox gene expressed in the developing brain. Mech Dev.

[47] Deschet K, Bourrat F, Chourrout D, Joly JS (1998). Expression domains of the medaka (*Oryzias latipes*) *Ol-Gsh 1* gene are reminiscent of those of clustered and orphan homeobox genes. Dev Genes Evol.

[48] Hudson C, Lemaire P (2001). Induction of anterior neural fates in the ascidian *Ciona intestinalis*. Mech Dev.

[49] Putnam NH, Butts T, Ferrier DEK, Furlong RF, Hellsten U, Kawashima T, Robinson-Rechavi M, Shoguchi E, Terry A, Yu JK (2008). The amphioxus genome and the evolution of the chordate karyotype. Nature.

[50] Brooke NM, Garcia-Fernandez J, Holland PWH (1998). The ParaHox gene cluster is an evolutionary sister of the Hox gene cluster. Nature.

[51] Jonsson J, Carlsson L, Edlund T, Edlund H (1994). Insulin-Promoter-Factor-1 is required for pancreas development in mice. Nature.

[52] Young T, Deschamps J, Pourquie O (2009). Hox, Cdx, and anteroposterior patterning in the mouse embryo. Hox genes.

[53] Corbo JC, Levine M, Zeller RW (1997). Characterization of a notochord-specific enhancer from the *Brachyury* promoter region of the ascidian, *Ciona intestinalis*. Development.

[54] Yu JK, Holland ND, Holland LZ (2004). Tissue-specific expression of *FoxD* reporter constructs in amphioxus embryos. Dev Biol.

[55] Beaster-Jones L, Schubert M, Holland LZ (2007). Cis-regulation of the amphioxus *engrailed* gene: insights into evolution of a muscle-specific enhancer. Mech Dev.

[56] Holland LZ, Albalat R, Azumi K, Benito-Gutiérrez È, Blow MJ, Bronner-Fraser M, Brunet F, Butts T, Candiani S, Dishaw LJ (2008). The amphioxus genome illuminates vertebrate origins and cephalochordate biology. Genome Res.

[57] Wada H, Kobayashi M, Zhang SC (2005). Ets identified as a trans-regulatory factor of amphioxus *Hox2* by transgenic analysis using ascidian embryos. Dev Biol.

[58] Natale A, Sims C, Chiusano ML, Amoroso A, D’Aniello E, Fucci L, Krumlauf R, Branno M, Locascio A (2011). Evolution of anterior Hox regulatory elements among chordates. BMC Evol Biol.

[59] Ovcharenko I, Loots GG, Giardine BM, Hou MM, Ma J, Hardison RC, Stubbs L, Miller W (2005). Mulan: multiple-sequence local alignment and visualization for studying function and evolution. Genome Res.

[60] Tassy O, Dauga D, Daian F, Sobral D, Robin F, Khoueiry P, Salgado D, Fox V, Caillol D, Schiappa R (2010). The ANISEED database: digital representation, formalization, and elucidation of a chordate developmental program. Genome Res.

[61] Huang SF, Chen ZL, Huang GR, Yu T, Yang P, Li J, Fu YG, Yuan SC, Chen SW, Xu AL (2012). HaploMerger: reconstructing allelic relationships for polymorphic diploid genome assemblies. Genome Res.

[62] Huang SF, Chen ZL, Yan XY, Yu T, Huang GR, Yan QY, Pontarotti PA, Zhao HC, Li J, Yang P (2014). Decelerated genome evolution in modern vertebrates revealed by analysis of multiple lancelet genomes. Nat Commun.

[63] Frazer KA, Pachter L, Poliakov A, Rubin EM, Dubchak I (2004). VISTA: computational tools for comparative genomics. Nucleic Acids Res.

[64] Harafuji N, Keys DN, Levine M (2002). Genome-wide identification of tissue-specific enhancers in the *Ciona* tadpole. Proc Natl Acad Sci U S A.

[65] Di Gregorio A, Levine M (2002). Analyzing gene regulation in ascidian embryos: new tools for new perspectives. Differentiation.

[66] Wada H, Saiga H, Satoh N, Holland PWH (1998). Tripartite organization of the ancestral chordate brain and the antiquity of placodes: insights from ascidian Pax-2/5/8, Hox and Otx genesHox and Otx genes. Development.

[67] Holland LZ, Carvalho JE, Escriva H, Laudet V, Schubert M, Shimeld SM, Yu JK (2013). Evolution of bilaterian central nervous systems: a single origin?. Evodevo.

[68] Hinman VF, Degnan BM (1998). Retinoic acid disrupts anterior ectodermal and endodermal development in ascidian larvae and postlarvae. Dev Genes Evol.

[69] Katsuyama Y, Wada S, Yasugi S, Saiga H (1995). Expression of the labial group Hox gene *HrHox-1* and its alteration induced by retinoic acid in development of the ascidian *Halocynthia roretzi*. Development.

[70] Kanda M, Ikeda T, Fujiwara S (2013). Identification of a retinoic acid-responsive neural enhancer in the *Ciona intestinalis Hox1* gene. Dev Growth Differ.

[71] Satou Y, Imai KS, Satoh N (2002). Fgf genes in the basal chordate *Ciona intestinalis*. Dev Genes Evol.

[72] Bertrand V, Hudson C, Caillol D, Popovici C, Lemaire P (2003). Neural tissue in ascidian embryos is induced by FGF9/16/20, acting via a combination of maternal GATA and Ets transcription factors. Cell.

[73] Imai KS, Stolfi A, Levine M, Satou Y (2009). Gene regulatory networks underlying the compartmentalization of the *Ciona* central nervous system. Development.

[74] Darras S, Nishida H (2001). The BMP/CHORDIN antagonism controls sensory pigment cell specification and differentiation in the ascidian embryo. Dev Biol.

[75] Christiaen L, Stolfi A, Levine M (2010). BMP signaling coordinates gene expression and cell migration during precardiac mesoderm development. Dev Biol.

[76] Hino K, Satou Y, Yagi K, Satoh N (2003). A genome-wide survey of developmentally relevant genes in *Ciona intestinalis* - VI. Genes for Wnt, TGF beta, Hedgehog and JAK/STAT signaling pathways. Dev Genes Evol.

[77] Sasakura Y, Ogasawara M, Makabe KW (1998). *HrWnt-5*: a maternally expressed ascidian Wnt gene with posterior localization in early embryos. Int J Dev Biol.

[78] Islam A, Moly PK, Miyamoto Y, Kusakabe TG (2010). Distinctive expression patterns of Hedgehog pathway genes in the *Ciona intestinalis* larva: implications for a role of Hedgehog signaling in postembryonic development and chordate evolution. Zoolog Sci.

[79] Imai KS, Hino K, Yagi K, Satoh N, Satou Y (2004). Gene expression profiles of transcription factors and signaling molecules in the ascidian embryo: towards a comprehensive understanding of gene networks. Development.

[80] Holland LZ, Holland ND (1996). Expression of *AmphiHox-1* and *AmphiPax-1* in amphioxus embryos treated with retinoic acid: insights into evolution and patterning of the chordate nerve cord and pharynx. Development.

[81] Holland PWH, Garcia-Fernàndez J (1996). Hox genes and chordate evolution. Dev Biol.

[82] Lin H-C, Holland LZ, Holland ND (2006). Expression of the *AmphiTcf* gene in amphioxus: Insights into the evolution of the TCF/LEF gene family during vertebrate evolution. Dev Dyn.

[83] Schmidt M, Patterson M, Farrell E, Munsterberg A (2004). Dynamic expression of Lef/Tcf family members and beta-catenin during chick gastrulation, neurulation, and early limb development. Dev Dyn.

[84] Gregorieff A, Grosschedl R, Clevers H (2004). Hindgut defects and transformation of the gastrointestinal tract in *Tcf4*(−/−)/*Tcf1*(−/−) embryos. EMBO J.

[85] Korinek V, Barker N, Willert K, Molenaar M, Roose J, Wagenaar G, Markman M, Lamers W, Destree O, Clevers H (1998). Two members of the Tcf family implicated in Wnt/beta-catenin signaling during embryogenesis in the mouse. Mol Cell Biol.

[86] Brunner E, Peter O, Schweizer L, Basler K (1997). *Pangolin* encodes a *Lef-1* homologue that acts downstream of Armadillo to transduce the Wingless signal in *Drosophila*. Nature.

[87] Faro A, Boj SF, Ambrosio R, van den Broek O, Korving J, Clevers H (2009). *T-Cell Factor 4* (*tcf7l2*) is the main effector of Wnt signaling during zebrafish intestine organogenesis. Zebrafish.

[88] Galceran J, Farinas I, Depew MJ, Clevers H, Grosschedl R (1999). *Wnt3a(−/−)*-like phenotype and limb deficiency in *Lef1*(−/−)*Tcf1*(−/−) mice. Genes Dev.

[89] Ikeya M, Lee SMK, Johnson JE, McMahon AP, Takada S (1997). Wnt signalling required for expansion of neural crest and CNS progenitors. Nature.

[90] Deschamps J, van de Ven C (2012). Concerted involvement of Cdx/Hox genes and Wnt signalling in morphogenesis of the caudal neural tube and cloacal derivatives from the posterior growth zone.

[91] Onai T, Lin HC, Schubert M, Koop D, Osborne PW, Alvarez S, Alvarez R, Holland ND, Holland LZ (2009). Retinoic acid and Wnt/beta-catenin have complementary roles in anterior/posterior patterning embryos of the basal chordate amphioxus. Dev Biol.

[92] McGregor AP, Pechmann M, Schwager EE, Feitosa NM, Kruck S, Aranda M, Damen WGM (2008). *Wnt8* is required for growth-zone establishment and development of opisthosomal segments in a spider. Curr Biol.

[93] McGregor AP, Pechmann M, Shwager EE, Damen WGM (2009). An ancestral regulatory network for posterior development in arthropods. Commun Integr Biol.

[94] Martin BL, Kimelman D (2009). Wnt signaling and the evolution of embryonic posterior development. Curr Biol.

[95] Chipman AD, Ferrier DEK, Brena C, Qu JX, Hughes DST, Schroder R, Torres-Oliva M, Znassi N, Jiang HY, Almeida FC (2014). The first myriapod genome sequence reveals conservative arthropod gene content and genome organisation in the centipede *Strigamia maritima*. PLoS Biol.

[96] Cole AG, Rizzo F, Martinez P, Fernandez-Serra M, Arnone MI (2009). Two ParaHox genes, *SpLox* and *SpCdx*, interact to partition the posterior endoderm in the formation of a functional gut. Development.

[97] Schubert M, Holland LZ, Holland ND (2000). Characterization of two amphioxus Wnt genes (*AmphiWnt4* and *AmphiWnt7b*) with early expression in the developing central nervous system. Dev Dyn.

[98] Schubert M, Holland LZ, Stokes MD, Holland ND (2001). Three amphioxus Wnt genes (*AmphiWnt3*, *AmphiWnt5*, and *AmphiWnt6*) associated with the tail bud: The evolution of somitogenesis in chordates. Dev Biol.

[99] Hobmayer B, Rentzsch F, Kuhn K, Happel CM, von Laue CC, Snyder P, Rothbacher U, Holstein TW (2000). WNT signalling molecules act in axis formation in the diploblastic metazoan *Hydra*. Nature.

[100] Kusserow A, Pang K, Sturm C, Hrouda M, Lentfer J, Schmidt HA, Technau U, von Haeseler A, Hobmayer B, Martindale MQ (2005). Unexpected complexity of the Wnt gene family in a sea anemone. Nature.

[101] Lapebie P, Gazave E, Ereskovsky A, Derelle R, Bezac C, Renard E, Houliston E, Borchiellini C (2009). WNT/beta-Catenin signalling and epithelial patterning in the homoscleromorph sponge *Oscarella*. PLoS One.

[102] Adamska M, Degnan SM, Green KM, Adamski M, Craigie A, Larroux C, Degnan BM (2007). Wnt and TGF-beta expression in the sponge *Amphimedon queenslandica* and the origin of metazoan embryonic patterning. PLoS One.

[103] Adamska M, Larroux C, Adamski M, Green K, Lovas E, Koop D, Richards GS, Zwafink C, Degnan BM (2010). Structure and expression of conserved Wnt pathway components in the demosponge *Amphimedon queenslandica*. Evol Dev.

[104] Adell T, Nefkens I, Muller WEG (2003). Polarity factor ‘Frizzled’ in the demosponge *Suberites domuncula*: identification, expression and localization of the receptor in the epithelium/pinacoderm. FEBS Lett.

[105] Adell T, Thakur AN, Mueller WEG (2007). Isolation and characterization of Wnt pathway-related genes from Porifera. Cell Biol Int.

[106] Fortunato SAV, Adamski M, Ramos OM, Leininger S, Liu J, Ferrier DEK, Adamska M (2014). Calcisponges have a ParaHox gene and dynamic expression of dispersed NK homeobox genes. Nature.

[107] Ramos OM, Barker D, Ferrier DEK (2012). Ghost loci imply Hox and ParaHox existence in the last common ancestor of animals. Curr Biol.

[108] Ferrier DEK, Dewar K, Cook A, Chang JL, Hill-Force A, Amemiya C (2005). The chordate ParaHox cluster. Curr Biol.

[109] Zeller RW, Virata MJ, Cone AC (2006). Predictable mosaic transgene expression in ascidian embryos produced with a simple electroporation device. Dev Dyn.

[110] Mayor C, Brudno M, Schwartz JR, Poliakov A, Rubin EM, Frazer KA, Pachter LS, Dubchak I (2000). VISTA: visualizing global DNA sequence alignments of arbitrary length. Bioinformatics.

[111] Farre D, Roset R, Huerta M, Adsuara JE, Rosello L, Alba MM, Messeguer X (2003). Identification of patterns in biological sequences at the ALGGEN server: PROMO and MALGEN. Nucleic Acids Res.

[112] Messeguer X, Escudero R, Farre D, Nunez O, Martinez J, Alba M (2002). PROMO: detection of known transcription regulatory elements using species-tailored searches. Bioinformatics.

[113] Wada S, Katsuyama Y, Yasugi S, Saiga H (1995). Spatially and temporally regulated expression of the LIM class homeobox gene *HRLIM* suggests multiple distinct functions in development of the ascidian, *Halocynthia roretzi*. Mech Dev.

