# Supplementary figures and images for: TCF/Lef regulates the Gsx ParaHox gene in central nervous system development in chordates
Source: BMC Evol Biol. 2016 Mar 3;16:57. doi: 10.1186/s12862-016-0614-3 (PMC4776371; doi:10.1186/s12862-016-0614-3)

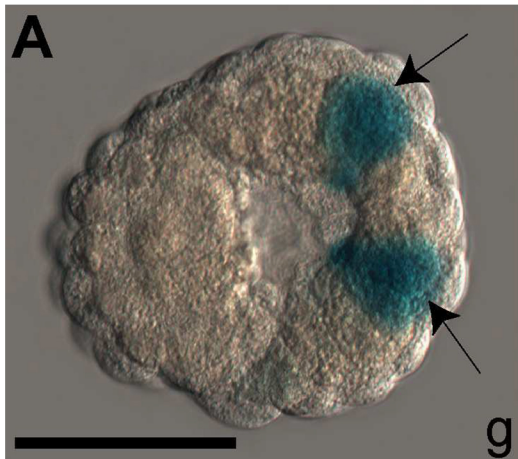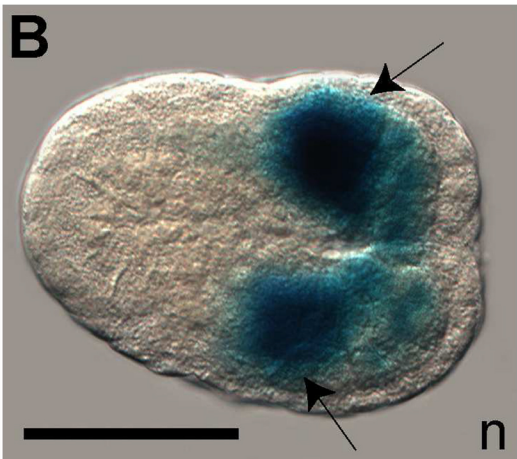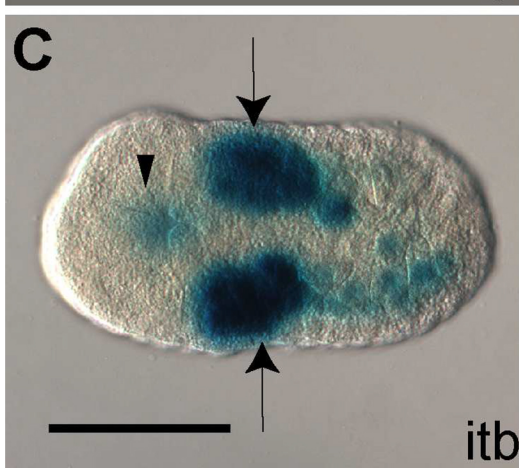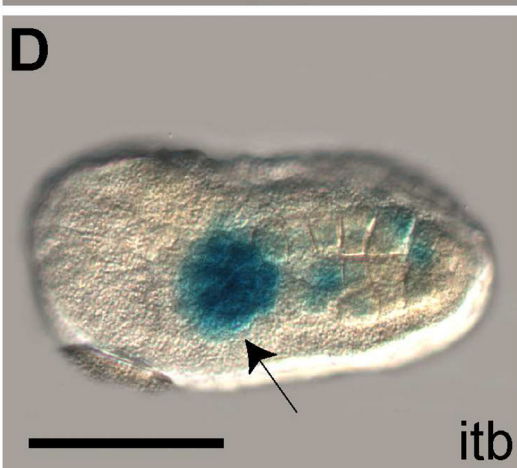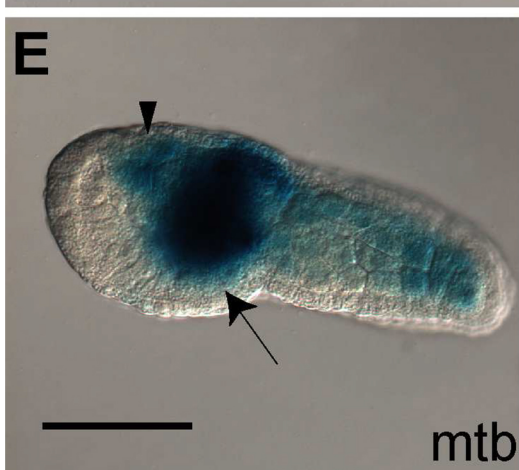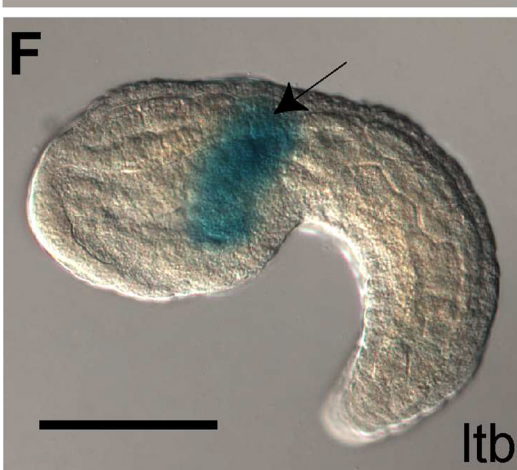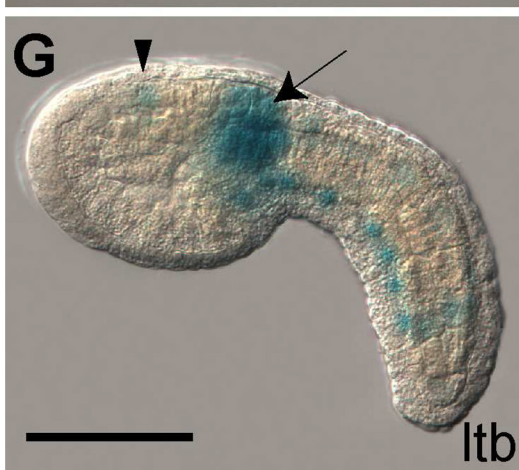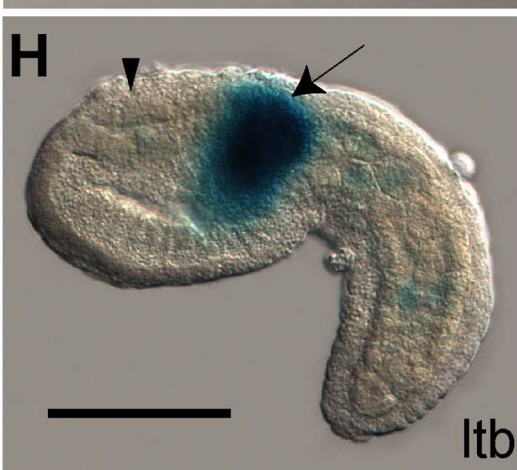

Supplement: Additional file 1: Figure S1. — Ectopic expression of pCES vector in Ciona intestinalis. Expression is almost always seen in mesenchymal tissue (A-H) (arrows). Expression is activated during gastrulation stages in mesenchymal cell lineages (A). At later stages, expression is visible posterior to the mesenchyme in variable numbers of tail muscle cells (C-H). Expression is also, though very rarely, observed in the centre of the sensory vesicle (black arrowheads) of tailbud stage embryos (A-C) show dorsal views, whilst (D-H) show lateral views. Lower case lettering refers to the stage of development; g, gastrula; n, neurula; itb, initial tailbud; mtb, mid tailbud; ltb, late tailbud. Scale bars represent 100 μm. (PDF 2350 kb) [file 12862_2016_614_MOESM1_ESM.pdf]
