# Supplementary material for: TCF/Lef regulates the Gsx ParaHox gene in central nervous system development in chordates
Source: BMC Evol Biol. 2016 Mar 3;16:57. doi: 10.1186/s12862-016-0614-3 (PMC4776371; doi:10.1186/s12862-016-0614-3)
Supplement: Additional file 2: Figure S2. — VISTA analysis of the polymorphic AmphiGsx-Upstream Proximal region. Sequences from multiple Branchiostoma floridae individuals, as well as from Branchiostoma belcheri were compared to the amphioxus ParaHox PAC sequence. The regions corresponding to the deletion constructs Gsx-Up3 and the 5′ half of Gsx-Up2 are the most variable, while the region covering Gsx-Up1 and the 3′ half of Gsx-Up2 is the most conserved. Note that Bf7_5 and Bf7_6 are different haplotypes from the same individual. Accession numbers for these sequences are found in the Methods. (PDF 1037 kb) [file 12862_2016_614_MOESM2_ESM.pdf]

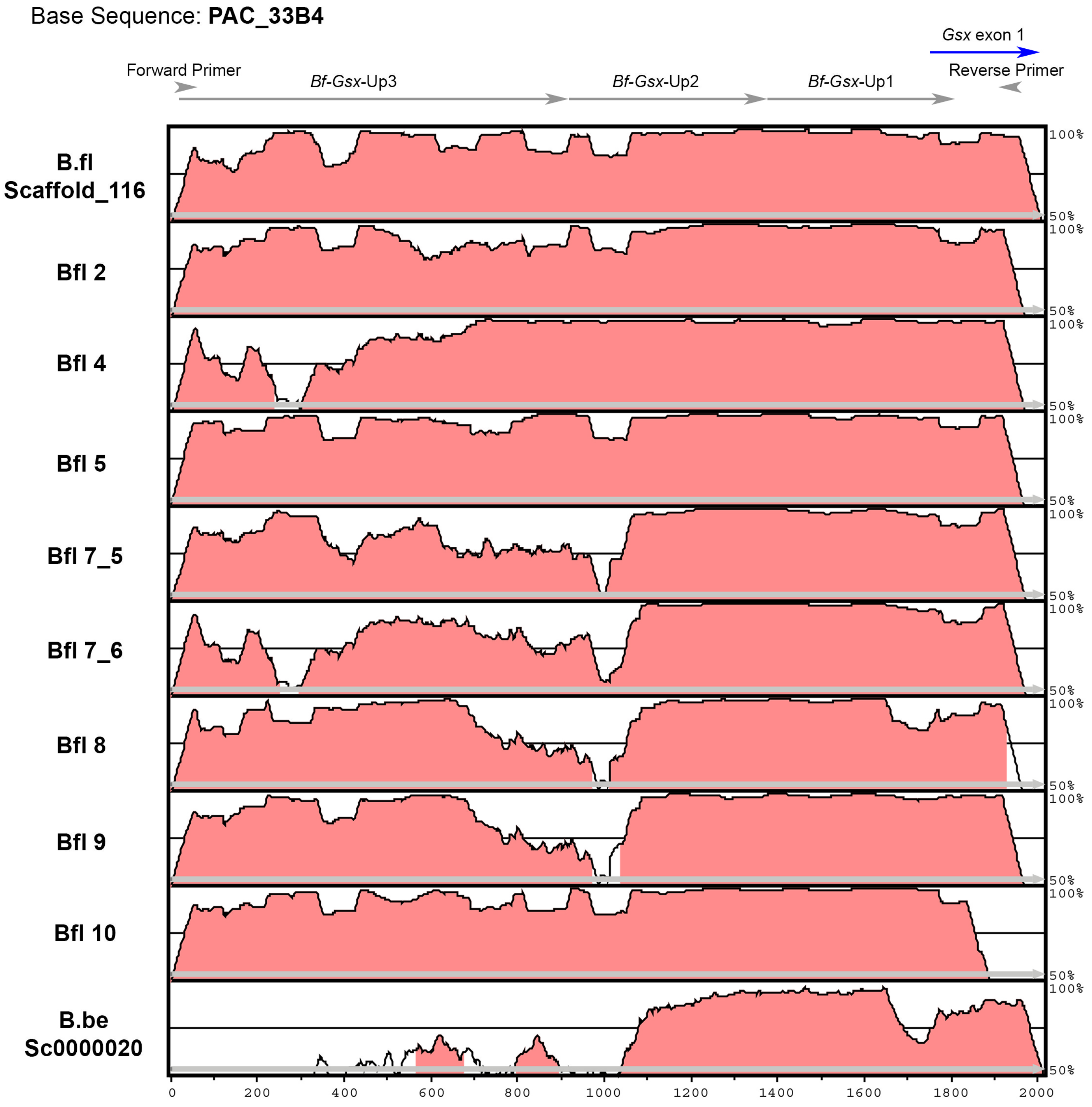

Base Sequence: **PAC_33B4**
Gsx exon 1
Forward Primer
Bf-Gsx-Up3
Bf-Gsx-Up2
Bf-Gsx-Up1
Reverse Primer
B.fl Scaffold_116
Bfl 2
Bfl 4
Bfl 5
Bfl 7_5
Bfl 7_6
Bfl 8
Bfl 9
Bfl 10
B.be Sc0000020
100%
50%
0
200
400
600
800
1000
1200
1400
1600
1800
2000
