# Supplementary material for: TCF/Lef regulates the Gsx ParaHox gene in central nervous system development in chordates
Source: BMC Evol Biol. 2016 Mar 3;16:57. doi: 10.1186/s12862-016-0614-3 (PMC4776371; doi:10.1186/s12862-016-0614-3)
Supplement: Additional file 3: Table S1. — Oligonucleotides used for cloning and mutagenesis. (DOCX 19 kb) [file 12862_2016_614_MOESM3_ESM.docx]

Supplementary Table 1

Oligonucleotides used for cloning and mutagenesis.

| Identifier | Sequence | Annealing Temp  (°C) | Primer  Modification |
| --- | --- | --- | --- |
| Bf-Gsx-up-poly-3Fa | GTGTCGGATGTTTGCCTTTT | 45 | n/a |
| Bf-Gsx-up-poly-1R | AAGTGGCTGTGTCCTGTGGT |  | n/a |
| Bf-Gsx-upF (Proximal) | GGATCCTGGGGGAAGAAGAACAA | 55 | BamHI  site |
| Bf-Gsx-upR (Proximal) | GGATCCCTTGAGTCGACTTCGGTGAC |  | BamHI  site |
| Bf-Gsx up3F | GCTGCAGAACGCAGCATACAA | 52 | PstI  Site |
| Bf-Gsx up3R | GCGGATCCACTTTGCCACCA |  | BamHI  Site |
| Bf Gsx up2F  (Gsx-Up2a) | CTGCAGTTGCATGGTGGCAAA | 52 | PstI  site |
| Bf-Gsx up 2aR  (Gsx-Up2a) | TGGGATCCAGGAGAAGGTAAACA |  | BamHI  site |
| Bf-Gsx up 2bF  (Gsx-Up2b) | CTGCAGCCATTCATGCCCGTT | 54 | PstI  site |
| Bf Gsx up2R  (Gsx-Up2b) | GGATCCAGTAGGAGTGAGGAC |  | BamHI  site |
| Bf Gsx up1F  (Gsx-Up1a) | CGCCTGCAGTCCTCACTCCTACT | 50 | PstI  site |
| BfGsxup 1aR  (Gsx-Up1a) | CTGCTCGGATCCTTTACTGCT |  | BamHI  site |
| BfGsxup 1bF  (Gsx-Up1b) | TGCTGCAGTAAAGGTCCGAGCAG | 50 | PstI  site |
| BfGsxup 1bR  (Gsx-Up1b) | TAGGATCCTTGAGTCGACTTCGGTGAC |  | BamHI  site |
| Bf-Gsx up1cF  (Gsx-Up1c) | CTGCAGAAAGGGCCTCTATTGCTTTC | 56 | PstI  site |
| Bf-Gsx up1cR  (Gsx-Up1c) | GGATCCAGCCCTTGCCAATGAAAAA |  | BamHI  site |
| TCF/Lef mut site1F | TGGCAAGAACTGAAAAATTGTTATTCCGTGTT | 70 | 5’ Phosphorylation |
| TCF/Lef mut site1R | CGCTTTTATACCTGCTCGGACCTTTACTGCTC |  | 5’ Phosphorylation |
| TCF/Lef mut site2F | CGTCGCGTCGAACGCAATTGTGAAGTCCACGT | 68 | 5’ Phosphorylation |
| TCF/Lef mut site2R | TATCTCTTCATTCGGTGCTGCATACAATTAAC |  | 5’ Phosphorylation |
| TCF/Lef mut site3F | AATCACTAAGGTAGGAATTGATGAAGTCTGCG | 70 | 5’ Phosphorylation |
| TCF/Lef mut site3R | ATCATTTATACTTGGAAGACATCGTTTCACGG |  | 5’ Phosphorylation |
| Ci-TCF/Lef F | CAGAGATTCCAGCCACAGAAGT | 58 | n/a |
| Ci-TCF/Lef R | TGGTTTCTTCACATATGGCCGA |  | n/a |

Names of oligonucleotides used within the study for cloning and mutagenesis are included, along with the corresponding oligonucleotide sequence, annealing temperatures used for primer pairs, and primer modifications for each oligonucleotide.
